# Evaluation of seasonal malaria chemoprevention in two areas of intense seasonal malaria transmission: Secondary analysis of a household-randomised, placebo-controlled trial in Houndé District, Burkina Faso and Bougouni District, Mali

**DOI:** 10.1371/journal.pmed.1003214

**Published:** 2020-08-21

**Authors:** Matthew E. Cairns, Issaka Sagara, Issaka Zongo, Irene Kuepfer, Ismaila Thera, Frederic Nikiema, Modibo Diarra, Serge R. Yerbanga, Amadou Barry, Amadou Tapily, Samba Coumare, Paul Milligan, Halidou Tinto, Jean Bosco Ouédraogo, Daniel Chandramohan, Brian Greenwood, Abdoulaye Djimde, Alassane Dicko

**Affiliations:** 1 Tropical Epidemiology Group, London School of Hygiene and Tropical Medicine, London, United Kingdom; 2 Malaria Research and Training Centre, Bamako, Mali; 3 Institut de Recherche en Sciences de la Santé, Bobo Dioulasso, Burkina Faso; 4 Swiss Tropical and Public Health Institute, Basel, Switzerland; 5 Faculty of Infectious and Tropical Diseases, London School of Hygiene and Tropical Medicine, London, United Kingdom; Mahidol University, THAILAND

## Abstract

**Background:**

Seasonal malaria chemoprevention (SMC) is now widely deployed in the Sahel, including several countries that are major contributors to the global burden of malaria. Consequently, it is important to understand whether SMC continues to provide a high level of protection and how SMC might be improved. SMC was evaluated using data from a large, household-randomised trial in Houndé, Burkina Faso and Bougouni, Mali.

**Methods and findings:**

The parent trial evaluated monthly SMC plus either azithromycin (AZ) or placebo, administered as directly observed therapy 4 times per year between August and November (2014–2016). In July 2014, 19,578 children aged 3–59 months were randomised by household to study group. Children who remained within the age range 3–59 months in August each year, plus children born into study households or who moved into the study area, received study drugs in 2015 and 2016. These analyses focus on the approximately 10,000 children (5,000 per country) under observation each year in the SMC plus placebo group. Despite high coverage and high adherence to SMC, the incidence of hospitalisations or deaths due to malaria and uncomplicated clinical malaria remained high in the study areas (overall incidence rates 12.5 [95% confidence interval (CI): 11.2, 14.1] and 871.1 [95% CI: 852.3, 890.6] cases per 1,000 person-years, respectively) and peaked in July each year, before SMC delivery began in August. The incidence rate ratio comparing SMC within the past 28 days with SMC more than 35 days ago—adjusted for age, country, and household clustering—was 0.13 (95% CI: 0.08, 0.20), P < 0.001 for malaria hospitalisations and deaths from malaria and 0.21 (95% CI 0.20, 0.23), P < 0.001 for uncomplicated malaria, indicating protective efficacy of 87.4% (95% CI: 79.6%, 92.2%) and 78.3% (95% CI: 76.8%, 79.6%), respectively. The prevalence of malaria parasitaemia at weekly surveys during the rainy season and at the end of the transmission season was several times higher in children who missed the SMC course preceding the survey contact, and the smallest prevalence ratio observed was 2.98 (95% CI: 1.95, 4.54), P < 0.001. The frequency of molecular markers of sulfadoxine-pyrimethamine (SP) and amodiaquine (AQ) resistance did not increase markedly over the study period either amongst study children or amongst school-age children resident in the study areas. After 3 years of SMC deployment, the day 28 PCR-unadjusted adequate clinical and parasitological response rate of the SP + AQ regimen in children with asymptomatic malaria was 98.3% (95% CI: 88.6%, 99.8%) in Burkina Faso and 96.1% (95% CI: 91.5%, 98.2%) in Mali. Key limitations of this study are the potential overdiagnosis of uncomplicated malaria by rapid diagnostic tests and the potential for residual confounding from factors related to adherence to the monthly SMC schedule.

**Conclusion:**

Despite strong evidence that SMC is providing a high level of protection, the burden of malaria remains substantial in the 2 study areas. These results emphasise the need for continuing support of SMC programmes. A fifth monthly SMC course is needed to adequately cover the whole transmission season in the study areas and in settings with similar epidemiology.

**Trial registration:**

The AZ-SMC trial in which these data were collected was registered at clinicaltrials.gov: NCT02211729.

## Abstract 2

Matthew Cairns and colleagues show additional courses of seasonal chemoprevention are needed in Burkina Faso and Mali to control malaria.

## Author summary

**Why was this study done?:**

- Seasonal malaria chemoprevention (SMC) is recommended for children under 5 years of age in countries of the Sahel and sub-Sahel. Many countries in West and Central Africa now have large-scale SMC programmes.
- The malaria burden remains high in several countries that have introduced SMC, including Burkina Faso and Mali, which are amongst the most important contributors to the global burden of malaria cases and deaths.
- It is important to understand whether SMC retains a high level of protection in these areas and how its impact might be improved.

**What did the researchers do and find?:**

- In this study, the level of protection provided by SMC was investigated using data from a large but closely supervised clinical trial in 2 districts in southern Burkina Faso and Mali.
- SMC was delivered 4 times per year over 3 years, reaching a very high percentage of children. All the daily doses of SMC were supervised by the study team. Specific substudies showed that molecular markers of resistance to the combination of antimalarials used for SMC were rare amongst malaria parasites and that the SMC combination was highly effective in curing infections detected at the end of the rainy season.
- Malaria incidence was markedly reduced in the period immediately after each SMC course. In the first 4 weeks after SMC, malaria cases were reduced by 78%, and malaria hospitalisations and deaths from malaria were reduced by 87%.
- Despite the benefits of SMC, the number of malaria cases, hospital admissions for malaria, and deaths from malaria remained very high in the study areas. There was a large peak in July each year, coinciding with the beginning of the rainy season, before SMC delivery began in August.

**What do these findings mean?:**

- SMC is likely to be averting a very large number of malaria cases, hospitalisations, and deaths in the study areas, but malaria has still not been brought under control.
- At least one additional monthly course of SMC is needed to address the high burden of malaria, including malaria deaths, that currently occurs outside the peak transmission season. This is likely to be the case in other areas of the Sahel that have a longer transmission season than can be covered by a 4-month SMC programme.
- Additional new tools are needed urgently to further reduce malaria in these districts and areas with similar epidemiology.

## Introduction

Seasonal malaria chemoprevention (SMC) comprises monthly courses of sulfadoxine-pyrimethamine plus amodiaquine (SP + AQ) given to children 3–59 months of age. After encouraging results from trials in a range of settings, including in the context of high coverage of long-lasting insecticide-treated nets (LLINs) [1,2], SMC became a WHO policy in 2012 for the prevention of malaria in areas of seasonal transmission [3,4], and it is now implemented widely in the Sahel and sub-Sahelian regions of Africa [5]. The current WHO recommendation specifies that SMC can be administered up to 4 times each year, although 5 monthly courses of SMC were deployed in South East Senegal and were highly effective and safe [6].

Evaluation of SMC at scale suggests that this intervention has an important impact on uncomplicated malaria, hospital admissions for malaria, and malaria deaths (the ACCESS-SMC partnership, submitted) [7–11]. However, despite the benefits of SMC, countries where SMC is currently deployed include some of the largest contributors to the global malaria burden, including northern Nigeria, Burkina Faso, and Mali [12]. In these areas of the Sahel and sub-Sahel, the malaria burden has remained very high despite scale-up of access to LLINs, effective artemisinin-based combination therapies (ACTs), and more recently SMC [12]. In such areas, it is unclear to what extent this represents ‘operational gaps’ in SMC programmes (e.g., deficiencies in coverage of the monthly courses or adherence to the 3-day regimen) and to what extent this is a ‘residual burden’ that would remain even if SMC was deployed under optimal conditions. This is an important distinction because any burden that would remain in the face of an optimised SMC programme must be tackled by other means. This could include adding monthly cycles of SMC to cover the burden that occurs outside the peak transmission season and providing SMC to older children, both of which have been shown to be effective and safe in Senegal [6,13], and, potentially, seasonally targeted vaccination to complement the protection from SMC [14].

Another concern that must be carefully monitored is the potential effect of drug resistance. Parasites resistant to SP or AQ could be at a selective advantage in areas where SMC is used widely, and this could lead to a reduction in the efficacy of SMC over time. Large-scale surveys of molecular markers have been conducted in several SMC countries [15]. Case–control studies have also been used to monitor the in vivo efficacy of SMC directly [16], but a range of other aspects need to be monitored to fully assess any potential changes in effectiveness.

In this study, data were reanalysed from a large-scale, closely supervised trial of SMC in southern Burkina Faso and Mali [17]. High coverage and adherence were achieved through directly observed treatment with SMC. The prevalence of molecular markers of parasite resistance to SP and AQ and the efficacy of the SMC regimen in clearing malaria parasitaemia were assessed. The high malaria incidence and prevalence in this context show the limitations of SMC in one of the epidemiological situations that is proving most challenging for malaria control.

## Methods

### Study procedures

This analysis uses data collected during the azithromycin (AZ)-SMC study, a household-randomised, placebo-controlled trial conducted in Houndé District, Burkina Faso and Bougouni District, Mali between August 2014 and December 2016 (S1 Fig), undertaken to investigate the potential value of adding AZ to the antimalarials used for SMC [17]. The primary outcome of the trial was hospital admissions and deaths; to investigate this outcome, a total sample size of at least 19,200 children was needed. In July 2014, 19,578 children (i.e., approximately 10,000 per country) aged 3–59 months were recruited through a household census of the study areas and randomised to intervention group by household. Children with a chronic disease, with a known allergy to SP, AQ, or AZ, or who were taking cotrimoxazole were not eligible for inclusion. The study was designed as an open cohort: children born into study households or who moved into the study area during the study period were eligible to receive SMC if aged between 3–59 months at the time of the first SMC course in August each year. Following national guidelines, children aged 60 months or more at the first SMC administration each year were no longer eligible. Children remained in follow-up until the study ended in December 2016 or the child died or exited the study area, was lost to follow-up, or became too old to receive SMC.

Between August and November each year, study children received 4 monthly courses of SMC with SP + AQ, with either AZ or a matching AZ placebo (hereafter SMC plus AZ and SMC plus placebo, respectively). SMC had not been deployed in the study area prior to the trial. In 2014, SMC delivery began in late August; in 2015 and 2016, delivery began on the first of August. Although there was no evidence of a benefit of AZ against all-cause hospitalisations and deaths, hospital admissions or deaths due to malaria, or uncomplicated clinical malaria, to avoid any possible influence of AZ on the results presented in this paper, only children who were randomised to SMC plus placebo are included. In total, 108,176 courses (321,642 individual daily doses) of SMC plus placebo were administered as directly observed therapy by the study team. All doses of study medication were recorded using tablet PCs. Scanning of the Quick Response (QR) code on the drug package and a child’s photo ID card were used to ensure linkage of treatment to the correct child, and the date and time stamp of each drug administration was recorded by the tablet PC.

Morbidity episodes were detected passively throughout the study period at the hospital and health centres in each study area (S2 and S3 Figs). In Mali, some children were treated by community health workers, using a rapid diagnostic test (RDT) to confirm *Plasmodium falciparum* parasitaemia. The child’s photo ID card was used to ensure that morbidity episodes were recorded for the correct study child. The following passively detected outcomes were included in this study: 1) incidence of malaria hospital admissions or deaths from malaria (as a combined outcome), defined as hospital admission for malaria combined with a positive blood slide or rapid diagnostic test, or deaths for which malaria was listed as the primary diagnosis; and 2) uncomplicated clinical malaria episodes, defined as history of fever or measured axillary temperature ≥37.5 °C combined with a positive RDT. Histidine-rich protein 2 (HRP2)-based RDTs were used to diagnose uncomplicated malaria and to guide treatment. Blood smears were taken from a systematic sample of study children for quality control of the RDT (slides were prepared 1 day per week in Burkina Faso and 1 week per month in Mali). All blood smears, both those used to diagnose malaria in hospital and those used for QC of the RDT, were double read, with discrepancies resolved by a third reader using a standardised algorithm [18]. For deaths occurring outside a health facility, verbal autopsies were used to ascertain cause of death, using the WHO Verbal Autopsy Questionnaire [19]. To assign a final diagnosis, both the verbal autopsy questionnaires and hospital records were reviewed independently by 2 physicians, with any disagreement reviewed by a third physician. The final diagnosis was reached by agreement between at least 2 physicians. All diagnoses were assigned prior to locking of the study database and breaking of the randomisation code. All reviewers were blind to the study group to which an individual child belonged.

At the end of each rainy season, the prevalence of *P*. *falciparum* infection was determined amongst randomly selected study children at cross-sectional surveys (target sample size of 2,000 per country, 1,000 per randomisation group in each country). Surveys were timed to occur at least 1 month after the final SMC administration and thus took place later in the first year of the study because of the later start in 2014. Two infection outcomes were defined: *P*. *falciparum* infection of any density and *P*. *falciparum* infection with a density ≥5,000 per microlitre. Blood samples were also taken through school-based surveys from school-age children resident in the same areas as the study children (500 per country in each year) who were not eligible to receive SMC because of their age.

The presence of molecular markers of resistance of *P*. *falciparum* to SP and AQ was determined amongst all study children who carried *P*. *falciparum* infection at the end-of-season surveys and from 50 school-age children with *P*. *falciparum* infection at the same time point. DNA was extracted from dried blood spots as described previously [20,21] (summarised in S1 Methods).

Each week during the malaria transmission season, *P*. *falciparum* prevalence was also determined amongst a sample of study children who were visited at home (i.e., active case detection). A target sample size of 100 per week (50 per randomisation group) was used in each country. Blood smears were taken and read later, so a positive result did not result in the child being treated. If a child had symptoms of malaria, they were tested with an RDT and, if positive, referred for treatment at the health centre.

### Ethical approval and consent

The AZ-SMC trial was approved by the ethics committees of the London School of Hygiene and Tropical Medicine, London (29 May 2013, no. 6355); the Malaria Research and Training Center, University of Bamako, Bamako, Mali (27 Feb 2014, no. 2014/16/CE/FMPOS); the Ministry of Health, Ouagadougou, Burkina Faso (20 March 2013, no. 2013-3-023); and the national regulatory authorities of Burkina Faso and Mali. Written informed consent was obtained from the parent or guardian of all study children.

### Analysis of the incidence of malaria hospital admissions or deaths from malaria and uncomplicated malaria

Person-time at risk was calculated from the first dose of SMC in 2014 (or from time of entry into the study for children born in study household or who migrated into the study area) until the study ended in December 2016 or the child died or exited the study area or became too old to receive SMC. For children lost to follow-up, the date of the last contact was used. Multiple events were included. Person-time was not adjusted after a malaria episode [22], but repeat contacts with healthcare for the same illness within 7 days of a prior episode were considered as a single event. Lexis expansion was used to stratify person-time for each child on the child’s current age in years, the calendar month, and the date of each SMC course.

The incidence of 1) malaria hospital admissions or deaths from malaria and 2) uncomplicated malaria was calculated by age in the 2 centres combined and by country. The incidence of these 2 outcomes was also calculated in each calendar month of the study, allowing comparison of periods when SMC was distributed (August–November) with periods when SMC was not deployed. For uncomplicated clinical malaria, incidence when SMC was deployed was further stratified according to whether a child had received SMC in the past 28 days (‘recent SMC’) or had missed the previous SMC course (‘no recent SMC’). To limit the potential for confounding by factors related to access to SMC, the incidence rates amongst children with ‘no recent SMC’ in each calendar month was restricted to children who otherwise received all SMC courses that year (i.e., received the other 3 of the 4 monthly SMC courses).

The detailed SMC history obtained for each child was used to further stratify person-time at risk according to the number of days since the most recent SMC course (0–7, 8–14, 15–21, 22–28, 28–35, >35 days, and no previous SMC in the current year). Person-time and events within these strata were then pooled over the study period to calculate incidence rates by time since SMC. Poisson regression with a gamma-distributed random effect fitted at the household level was used to calculate incidence rate ratios comparing incidence within the first 28 days post-SMC with incidence in the period beyond 35 days post-SMC (i.e., beyond the monthly delivery schedule), accounting for household clustering and adjusting for age of the child in years.

### Analysis of the prevalence of *P*. *falciparum* infection

For the end-of-season surveys, the prevalence of *P*. *falciparum* infection was calculated for study children and for school-age children resident in the study areas. Prevalence was also calculated separately for study children who had and had not received SMC at the most recent round of SMC administration before the survey. Prevalence ratios were calculated comparing children with and without recent SMC using modified Poisson regression [23], with a robust standard error to account for household clustering and adjusting for age of the child in years.

For the weekly surveys during the transmission season, *P*. *falciparum* prevalence was calculated overall and according to receipt (or not) of the most recent round of SMC administration prior to the weekly survey. As for the end-of-season surveys, prevalence ratios were calculated comparing children with and without recent SMC using modified Poisson regression [23], with a robust standard error to account for the household-randomised design and adjusting for age of the child in years.

### Analysis of the frequency and prevalence of molecular markers of SP and AQ resistance

The frequency and prevalence of resistant mutants were calculated as 1) the number of resistant mutants/the total number of clones and 2) the number of resistant mutants/the number of study children, respectively (further details are provided in S1 Methods). For prevalence, infections carrying mixed mutations at a single locus were counted as resistant. Samples for which 2 or more codons had both mutant and wild type present were excluded from the analysis.

### Analysis of the treatment efficacy of SP + AQ

At the end of the study period, after 3 years of SMC deployment in each of the study areas, a treatment efficacy study was undertaken amongst children with asymptomatic *P*. *falciparum* infection at the final cross-sectional survey. Children with no symptoms of malaria with a positive RDT, subsequently confirmed by microscopy, were eligible for this study. Fifty-eight and 153 children were included in Burkina Faso and Mali, respectively. These children received a full treatment course of SP + AQ over 3 days, dosed according to age, i.e., the same regimen as used for SMC. Children were then followed for 28 days according to a standard treatment efficacy protocol, with parasitaemia and symptoms assessed at days 1, 2, 4, 7, 14, 21, and 28 after treatment. Infections after day 7 (late parasitological failures) were investigated to determine whether the infection was caused by a recrudescence or by reinfection. DNA extracted from dried blood spots using a previously published method (described in the S1 Methods) were used for molecular analyses of *msp1*, *msp2*, and *CA1* polymorphisms to distinguish recrudescent from new infections. Cases of post-treatment mixed infection that contained parasites from day 0 were counted as recrudescent. Change of profile in any or both loci was counted as reinfection. Adequate clinical and parasitological response (ACPR) rates were recalculated both unadjusted for reinfection and adjusted for reinfection by limiting the cases of failure to true recrudescent parasites [24].

### Statistical analysis plan

Analysis of the frequency and prevalence of molecular markers of SP and AQ resistance amongst study children and school-age children and the treatment efficacy of SP plus AQ amongst children with asymptomatic *P*. *falciparum* infection were prespecified in the statistical analysis plan for the main trial [17]. Analyses of malaria incidence by age, by calendar time, and by time since the most recent SMC administration and analysis of prevalence according to the time since most recent SMC administration were not included in the trial’s statistical analysis plan, but these secondary analyses were planned prior to breaking the study randomisation code, and the locked databases were used for all analyses.

## Results

In the first year of the study (August 2014–July 2015), 9,603 children were under observation in the placebo group. The cohort under observation grew to 10,760 and 11,047 children in the second and third years, respectively, with similar numbers in each country throughout the study. Demographic information is presented in Table 1. The cohort was approximately 51% male throughout the study period and increased slightly in age in successive years (mean age 2.01 in year 1, 2.14 in year 2, and 2.17 in year 3). LLIN use was high in both countries prior to enrolment (72.4% in Burkina Faso and 72.7% in Mali), after which all children received a new LLIN from the study team.

**Table 1:** Characteristics of children in follow-up in each year of the study.

	Burkina Faso		Mali		Both Centres	
	No.	%	No.	%	No.	%
**Follow-up in Study Year 1 (2014)**						
Number of children in placebo group	4,758		4,845		9,603	
Age in years on August 1st, 2014						
0	909	19.1	830	17.1	1,739	18.1
1	1,013	21.3	991	20.5	2,004	20.9
2	960	20.2	1,084	22.4	2,044	21.3
3	966	20.3	1,061	21.9	2,027	21.1
4	910	19.1	879	18.1	1,789	18.6
Mean age in years (SD)	1.99	1.39	2.03	1.35	2.01	1.37
Sex						
Male	2,471	51.9	2,462	50.8	4,933	51.4
LLIN use prior to study*						
yes	3,428	72.4	3,305	72.7	6,733	72.5
missing	26		296		322	
**Follow-up in Study Year 2 (2015)**						
Number of children in placebo group	5,324		5,436		10,760	
Age in years on August 1st, 2015						
0	805	15.1	703	12.9	1,508	14
1	1,165	21.9	1,156	21.3	2,321	21.6
2	1,166	21.9	1,203	22.1	2,369	22
3	1,090	20.5	1,240	22.8	2,330	21.7
4	1,098	20.6	1,134	20.9	2,232	20.7
Mean age in years (SD)	2.10	1.36	2.17	1.33	2.14	1.34
Sex						
Male	2,761	51.9	2,742	50.5	5,503	51.2
**Follow-up in Study Year 3 (2016)**						
Number of children in placebo group	5,423		5,624		11,047	
Age in years on August 1st, 2016						
0	751	13.8	805	14.3	1,556	14.1
1	1,120	20.7	1,078	19.2	2,198	19.9
2	1,208	22.3	1,261	22.4	2,469	22.3
3	1,212	22.3	1,232	21.9	2,444	22.1
4	1,132	20.9	1,248	22.2	2,380	21.5
Mean age in years (SD)	2.16	1.34	2.18	1.36	2.17	1.35
Sex						
Male	2,842	52.4	2,864	51	5,706	51.7

*Information on LLIN use was only collected in 2014 because all children were issued with a new LLIN upon enrolment. **Abbreviations:** LLIN, long-lasting insecticide-treated net.

Adherence to the monthly SMC schedule and 3-day SMC regimen were high in both countries (S1 Table and S2 Table). The percentage of study children receiving all 4 monthly courses of SMC ranged from 64.9% to 75.4% in Burkina Faso and between 61.6% and 74.3% in Mali. The percentage of study children who received at least 3 cycles was 86.0% to 93.8% in Burkina Faso and 82.3% to 91.1% in Mali. Amongst children receiving the first dose of each cycle, the number who received all 3 daily doses ranged from 94.7% to 99.9% in Burkina Faso and 91.2% to 99.3% in Mali.

### Incidence of malaria hospital admissions or deaths from malaria and uncomplicated clinical malaria

In total, 23,817.2 person-years of follow-up were recorded amongst children in the SMC plus placebo group (11,772.3 and 12,042.6 person-years in Burkina Faso and Mali, respectively). One hundred thirty-five nonfatal malaria hospital admissions, 28 deaths from malaria, and 10,329 uncomplicated clinical malaria episodes occurred amongst children in the SMC plus placebo group in Burkina Faso; the corresponding number of these events were 109, 26, and 10,419, respectively, in children randomised to SMC plus placebo in Mali. The overall incidence of malaria hospital admissions or death from malaria and uncomplicated malaria was 12.5 (95% confidence interval [CI]: 11.2, 14.1) and 871.1 (95% CI: 852.3, 890.6) cases per 1,000 child-years at risk, respectively. The rates were slightly higher in Burkina Faso: 13.8 (95% CI: 11.9, 16.2) malaria hospital admissions or deaths from malaria and 877.4 (95% CI: 852.3, 903.5) uncomplicated malaria episodes per 1,000 person-years at risk, whereas in Mali, the corresponding rates were 11.2 (95% CI: 9.45, 13.4) and 865.2 (95% CI: 837.4, 894.3) per 1,000 person-years. Amongst 2,626 episodes of uncomplicated malaria for which a blood smear was available in addition to the RDT result, 31.3% (95% CI: 28.9%, 33.8%) were slide-negative in Burkina Faso and 31.3% (95% CI: 28.4%, 34.4%) in Mali.

**Fig 1:**
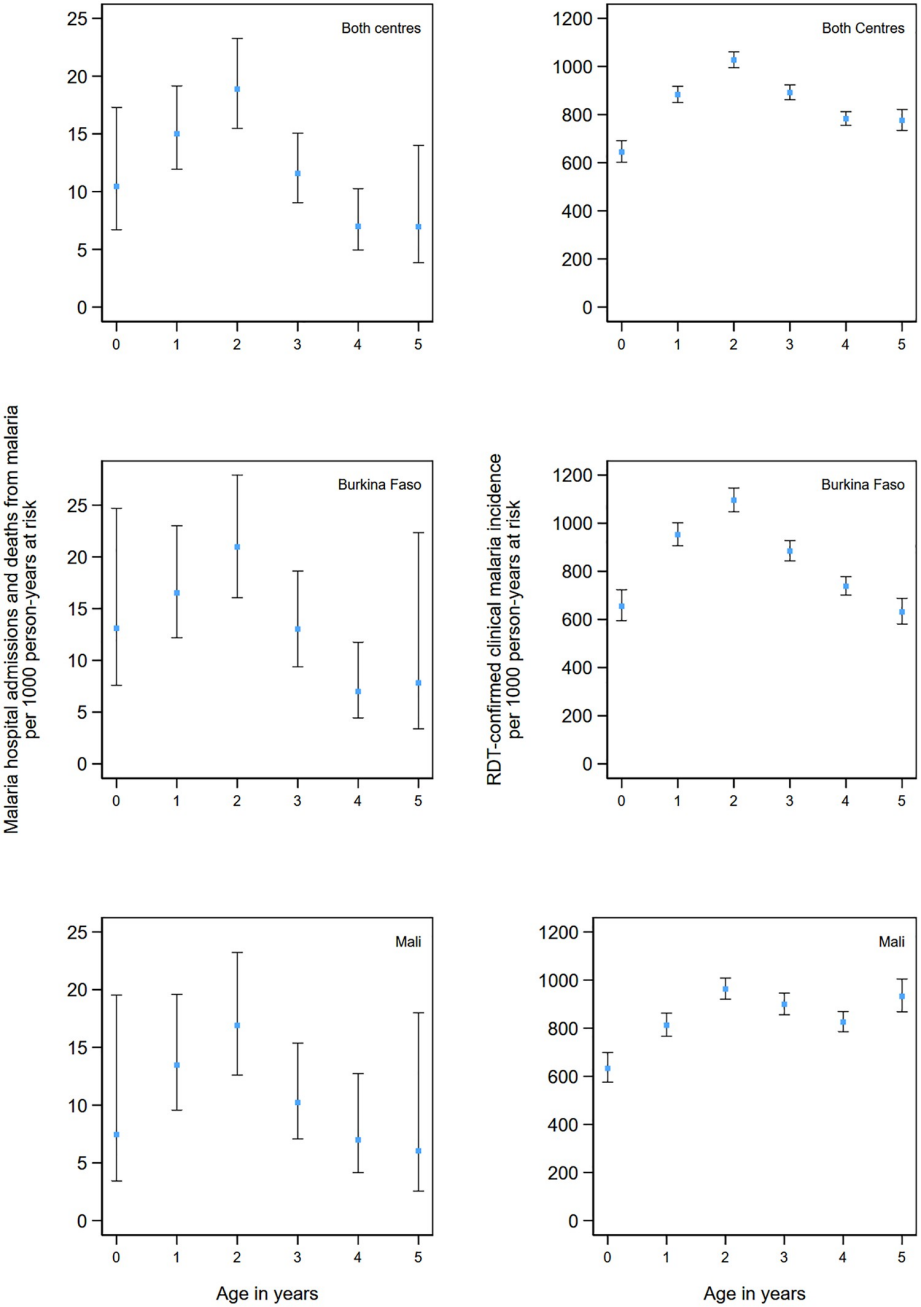
Incidence of malaria hospital admissions and deaths from malaria and clinical malaria by age. Incidence of malaria hospitalisations and deaths from malaria (left panel) and uncomplicated clinical malaria (right panel) by age group over the study period. Malaria hospitalisations and deaths from malaria were defined as hospital admission with a diagnosis of malaria and blood-slide–or RDT-confirmed *P*. *falciparum* infection or deaths for which malaria was listed as the primary diagnosis. Clinical malaria was defined as attendance at study health facility with a history of fever or measured temperature ≥37.5 °C, with malaria infection confirmed by RDT. Incidence rates are presented per 1,000 person-years and include repeat events in the same child, provided the healthcare contact occurred more than 7 days apart. Vertical bars show 95% CIs. CI, confidence interval; RDT, rapid diagnostic test.

Incidence of both hospital admissions and deaths from malaria and uncomplicated malaria peaked in children 2 years of age in both countries (Fig 1). Malaria deaths specifically peaked in infancy in Burkina Faso and in 1- and 2-year old children in Mali, although the number of deaths in each year of age were low, and thus, CIs are wide (S4 Fig).

#### Analysis by calendar month

**Fig 2:**
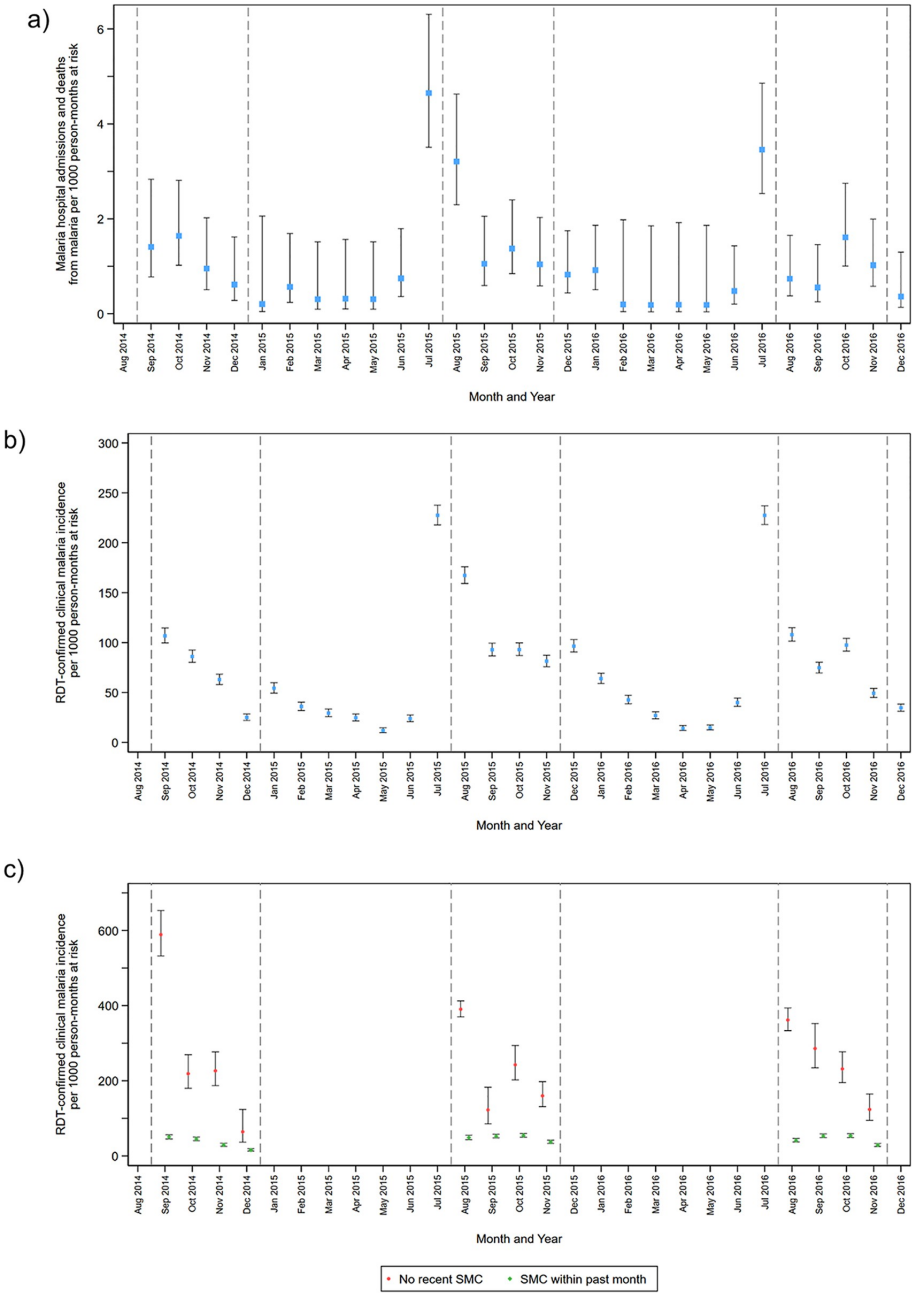
The incidence of malaria hospitalisations and deaths from malaria and uncomplicated clinical malaria over the study period by month of year. Incidence of malaria hospitalisations and deaths from malaria (a) and clinical malaria (b) by calendar month over the study period. (c) shows the incidence of uncomplicated malaria during the period when SMC was delivered (shown by vertical dashed lines) amongst children who had received SMC within the previous 28 days or who had not received recent SMC (no SMC in the previous 35 days). Incidence rates are presented as per 1,000 person-months at risk rather than per 1,000 person-years and include repeat events in the same child, provided the healthcare contact occurred more than 7 days apart. Vertical bars show 95% CIs. The analysis of children with ‘no recent SMC’ was restricted to children who received 3 courses of SMC during that intervention year (i.e., this excludes children who missed SMC on more than one occasion). Malaria hospitalisations and deaths from malaria were defined as hospital admission with a diagnosis of malaria and blood-slide–or RDT-confirmed *P*. *falciparum* infection or deaths for which malaria was listed as the primary diagnosis. Clinical malaria was defined as attendance at study health facility with a history of fever or measured temperature ≥37.5 °C, with malaria infection confirmed by RDT. CI, confidence interval; RDT, rapid diagnostic test; SMC, seasonal malaria chemoprevention.

In each year of the study, both malaria hospitalisations and deaths from malaria (Fig 2a) and uncomplicated clinical malaria (Fig 2b) peaked in July, prior to delivery of the first SMC course in August. During the rainy season, when SMC was given, the incidence of malaria hospitalisations and deaths from malaria remained slightly higher than during the dry season period but was markedly lower than the incidence immediately before SMC delivery began. Despite high SMC coverage, the overall incidence of uncomplicated malaria diagnosed by RDT was still 100 cases per 1,000 child-months during the rainy season. During the peak transmission period, the incidence of uncomplicated clinical malaria was very low amongst children with recent receipt of SMC, whereas children without recent SMC had a much higher incidence (Fig 2c). These results were similar in the 2 study sites (S5 and S6 Figs), with a slightly higher incidence in Burkina Faso.

#### Analysis by time since SMC

**Fig 3:**
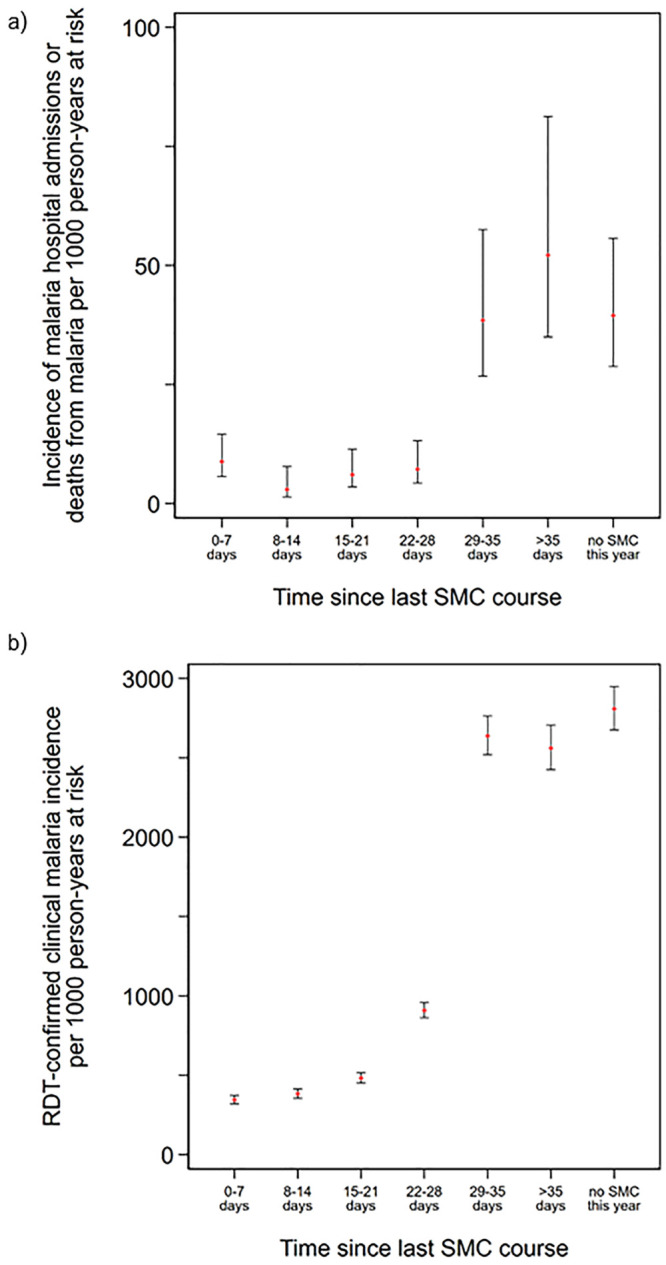
The incidence of malaria hospitalisations and deaths from malaria and episodes of uncomplicated clinical malaria by time since the most recent SMC treatment. Incidence of malaria hospitalisations and deaths from malaria (a) and clinical malaria (b) according to time since the most recent SMC course. Incidence rates are presented per 1,000 person-years at risk. Vertical bars show 95% CIs. ‘No SMC this year’ indicates children who had not yet received their first course of SMC in that year of the study (but who subsequently received SMC). Malaria hospitalisations and deaths from malaria were defined as hospital admission with a diagnosis of malaria and blood-slide–or RDT-confirmed *P*. *falciparum* infection or deaths for which malaria was listed as the primary diagnosis. Clinical malaria was defined as attendance at study health facility with a history of fever or measured temperature ≥37.5 °C, with malaria infection confirmed by RDT. CI, confidence interval; RDT, rapid diagnostic test; SMC, seasonal malaria chemoprevention.

The incidence of malaria hospitalisations and deaths from malaria was 13.6 per 1,000 child-years at risk (95% CI: 11.2, 16.8) in the first 28 days after SMC treatment (Fig 3a) but increased markedly beyond this time period to 51.1 per 1,000 child-years (95% CI: 42.1, 62.7). The incidence rate ratio comparing SMC within the past 28 days to SMC more than 35 days ago (i.e., not adherent to the monthly schedule) was 0.13 (95% CI: 0.08, 0.20), P < 0.001, indicating a protective efficacy of 87.4% (95% CI: 79.6%, 92.2%).

A similar pattern was observed for the incidence of uncomplicated clinical malaria (Fig 3b), with a low incidence rate in the first 3 weeks after SMC (402.1 [95% CI: 383.5, 421.9] overall in the first 21 days). This increased to 908.4 cases per 1,000 child-years (95% CI: 862.3, 957.7) between 22 to 28 days since last receipt of SMC and then exceeded 2,500 cases per 1,000 child-years at risk from 29 days post-SMC onwards. The incidence rate ratio comparing SMC within the past 28 days to SMC more than 35 days ago was 0.21 (95% CI 0.20, 0.23), P < 0.001, indicating a protective efficacy of 78.3% (95% CI: 76.8%, 79.6%).

### Prevalence of *P*. *falciparum* at cross-sectional surveys

#### End-of-season surveys

The overall prevalence of *P*. *falciparum* amongst study children was below 10% at all 3 end-of-season cross-sectional surveys in both countries (Table 2). Infections with parasite density in excess of 5,000 per microlitre were found in less than 5% of study children in all but one survey (Mali, 2015, in which prevalence was 6.2%). Compared with children who had received the most recent SMC course, prevalence was markedly higher amongst study children who had missed the most recent SMC prior to the cross-sectional survey: the smallest prevalence ratio was 3.02 (95% CI: 1.98, 4.60), P < 0.001. Prevalence ratios remained comparable when children who missed the most recent SMC course were restricted to those who had otherwise received all SMC courses that year (S3 Table). Amongst school-age children, who were not eligible for SMC, the prevalence of *P*. *falciparum* parasitaemia of any density was above 50% in all 3 cross-sectional surveys in both countries.

**Table 2:** *P*. *falciparum* parasitaemia at the end of the malaria transmission season surveys amongst study children in the placebo group and in school-age children.

	All Children				Children Who Received Final SMC Cycle before the Survey		Children Who Missed the Final SMC Cycle before the Survey			
	Any parasitaemia		Parasitaemia ≥5,000/μl		Any parasitaemia		Any parasitaemia			
	n/N	% (95% CI)	n/N	% (95% CI)	n/N	% (95% CI)	n/N	% (95% CI)	Prevalence ratio* (95% CI)	P-value
**Burkina Faso**										
**Study Children (SMC plus Placebo Group)**										
2014	27/1,009	2.68 (1.84, 3.88)	5/1,009	0.50 (0.21, 1.19)	20/976	2.05 (1.33, 3.16)	7/33	21.2 (10.4, 38.3)	11.8 (5.68, 24.5)	<0.001
2015	97/983	9.87 (8.16, 11.9)	48/983	4.88 (3.68, 6.44)	77/920	8.37 (6.74, 10.4)	20/63	31.7 (21.5, 44.2)	3.60 (2.30, 5.63)	<0.001
2016	83/973	8.53 (6.90, 10.5)	35/973	3.60 (2.60, 4.96)	64/909	7.04 (5.51, 8.95)	19/64	29.7 (19.6, 42.2)	4.07 (2.57, 6.45)	<0.001
**School-Age Children**										
2014	308/497	62.0 (57.6, 66.1)	23/497	4.63 (3.09, 6.87)						
2015	324/529	61.2 (57.0, 65.3)	29/529	5.48 (3.83, 7.78)						
2016	251/500	50.2 (45.8, 54.6)	28/500	5.60 (3.89, 8.00)						
**Mali**										
**Study Children (SMC plus Placebo Group)**										
2014	57/988	5.77 (4.44, 7.47)	27/988	2.73 (1.89, 3.94)	49/950	5.16 (3.91, 6.78)	8/38	21.1 (10.4, 38.1)	4.80 (2.36, 9.76)	<0.001
2015	90/983	9.16 (7.46, 11.2)	61/983	6.21 (4.85, 7.92)	47/861	5.46 (4.13, 7.19)	36/109	33.0 (24.3, 43.2)	5.61 (3.77, 8.33)	<0.001
2016	90/1,009	8.92 (7.28, 10.9)	28/1,009	2.78 (1.95, 3.94)	67/906	7.40 (5.83, 9.34)	23/103	22.3 (15.4, 31.2)	2.98 (1.95, 4.54)	<0.001
**School-Age Children**										
2014	294/496	59.3 (54.9, 63.5)	31/496	6.25 (4.43, 8.76)						
2015	326/500	65.2 (60.9, 69.3)	47/500	9.40 (7.13, 12.3)						
2016	268/500	53.6 (49.2, 57.9)	26/500	5.20 (3.56, 7.53)						

Prevalence of *P*. *falciparum* parasitaemia at the end-of-season cross-sectional survey (carried out at least 1 month after the last SMC cycle in each year). The table shows infection of any density and density ≥5,000 per microlitre overall amongst study children in the SMC plus placebo group and according to receipt of SMC at the most recent delivery cycle. **Abbreviations:** CI, confidence interval; SMC, seasonal malaria chemoprevention.

*Prevalence ratios compare children who received the final SMC before the survey and who missed the final SMC course and adjust for age and household-level clustering. Prevalence amongst school-age children resident in the study area (data from Chandramohan and colleagues [17]) is included for comparative purposes.

#### Weekly surveys during the transmission season

Prevalence of *P*. *falciparum* parasitaemia at the weekly surveys (Table 3) was generally lower than at the end-of-season surveys because most children were protected by SMC at the time of sampling, and prevalence was below 10% in all 3 years in both countries. As was the case for the end-of-season surveys, children who had not received SMC in the past 28 days at the time of the weekly survey were at a much higher risk of carrying infection than those who had: the smallest prevalence ratio observed was 4.32 (95% CI: 2.46, 7.58), P < 0.001).

**Table 3:** Malaria parasitaemia at the weekly surveys in the SMC plus placebo group.

	All Children				SMC in Past 28 Days		No SMC in Past 28 Days			
	Any parasitaemia		Parasitaemia ≥5,000/μl		Any parasitaemia		Any parasitaemia			
	n/N	% (95% CI)	n/N	% (95% CI)	n/N	% (95% CI)	n/N	% (95% CI)	Prevalence ratio* (95% CI)	P-value
**Burkina Faso**										
2014	43/863	4.98 (3.71, 6.65)	15/863	1.74 (1.05, 2.86)	15/685	2.19 (1.32, 3.61)	28/177	15.8 (11.1, 22.0)	7.29 (3.96, 13.4)	<0.001
2015	59/792	7.45 (5.80, 9.53)	30/792	3.79 (2.66, 5.36)	26/618	4.21 (2.88, 6.11)	33/171	19.3 (14.0, 25.9)	4.47 (2.77, 7.22)	<0.001
2016	43/837	5.14 (3.81, 6.90)	19/951	2.00 (1.25, 3.18)	19/690	2.75 (1.72, 4.38)	24/146	16.4 (11.3, 23.3)	5.56 (3.09, 9.98)	<0.001
**Mali**										
2014	6/644	0.93 (0.42, 2.06)	2/644	0.31 (0.08, 1.24)	1/505	0.20 (0.03, 1.40)	5/139	3.60 (1.50, 8.39)	18.5 (2.31, 148.1)	0.006
2015	48/771	6.23 (4.73, 8.16)	22/771	2.85 (1.89, 4.29)	21/611	3.44 (2.26, 5.20)	23/155	14.8 (10.0, 21.4)	4.07 (2.31, 7.15)	<0.001
2016	38/761	4.99 (3.68, 6.75)	19/850	2.24 (1.44, 3.46)	13/579	2.25 (1.31, 3.83)	25/181	13.8 (9.52, 19.6)	6.42 (3.38, 12.2)	<0.001

Prevalence of *P*. *falciparum* parasitaemia at weekly survey contacts during the malaria transmission season (i.e., between the first SMC cycle in each year and 1 month after the final SMC cycle). The table shows *P*. *falciparum* infection of any density and density ≥5,000 per microlitre amongst study children overall and according to whether the child received SMC at the most recent delivery cycle prior to the survey. **Abbreviations:** CI, confidence interval; SMC, seasonal malaria chemoprevention.

*Prevalence ratios compare children who had received SMC within the past 28 days and those with no SMC in the past 28 days and adjust for age and household-level clustering.

### Molecular markers of resistance to SP and AQ

The frequency of molecular markers associated with AQ resistance (*P*. *falciparum* chloroquine resistance transporter, *pfcrt* K76T; *P*. *falciparum* multidrug resistance 1, *pfmdr1* N86Y; and the combined *pfcrt* K76T + *pfmdr1* N86Y haplotype) did not increase amongst study children between 2014 and 2016 (Table 4). Likewise, the frequency of molecular markers associated with both pyrimethamine resistance (*P*. *falciparum* dihydrofolate reductase, *pfdhfr*-C59R) and sulfadoxine resistance (*P*. *falciparum* dihydropteroate synthase, *pfdhps-*A437G and *pfdhps-*K540E) did not increase over the study period. In particular, the *pfdhps*-K540E mutation, associated with high-grade resistance to sulfadoxine, remained at low frequency, with just 8 out of 254 samples resistant in 2016. Similar results were observed by country or when the prevalence of resistance markers amongst study children was calculated (S4 Table).

**Table 4:** Frequency of molecular markers of SP and AQ resistance amongst study children in the placebo group overall and by study country.

	Overall		Burkina Faso		Mali	
Mutation	n/N	Frequency, % (95% CI)	n/N	Frequency, % (95% CI)	n/N	Frequency, % (95% CI)
**2014**						
*pfcrt* K76T	70/110	63.6 (54.5, 71.9)	15/36	41.7 (28.5, 56.2)	55/74	74.3 (63.1, 83.0)
*pfmdr1* N86Y	23/110	20.9 (14.6, 29.0)	8/36	22.2 (10.6, 40.9)	15/74	20.3 (13.4, 29.4)
*pfcrt* K76T + *pfmdr1* N86Y	17/110	15.5 (10.2, 22.7)	4/36	11.1 (4.22, 26.2)	13/74	17.6 (11.2, 26.6)
*pfdhfr* C59R	73/99	73.7 (63.3, 82.0)	29/33	87.9 (67.3, 96.2)	44/66	66.7 (53.7, 77.5)
*pfdhps* A437G	73/99	73.7 (64.6, 81.2)	23/33	69.7 (51.0, 83.6)	50/66	75.8 (65.3, 83.8)
*pfdhps* K540E	2/99	2.02 (0.50, 7.79)	1/33	3.03 (0.42, 19.0)	1/66	1.52 (0.20, 10.4)
*pfdhfr* + *pfdhps*-437	57/99	57.6 (48.3, 66.4)	21/33	63.6 (44.9, 79.0)	36/66	54.5 (43.7, 65.0)
*pfdhfr* + *pfdhps-*437 + *pfdhps*-540	0/99	0	0/33	0	0/66	0
**2016**						
*pfcrt* K76T	53/119	44.5 (35.2, 54.2)	7/59	11.9 (5.82, 22.7)	46/60	76.7 (65.0, 85.3)
*pfmdr1* N86Y	19/119	16.0 (10.6, 23.3)	8/59	13.6 (6.84, 25.1)	11/60	18.3 (10.8, 29.4)
*pfcrt* K76T + *pfmdr1* N86Y	10/119	8.40 (4.66, 14.7)	0/59	0	10/60	16.7 (9.55, 27.5)
*pfdhfr* C59R	115/130	88.5 (81.8, 92.9)	61/64	95.3 (86.7, 98.4)	54/66	81.8 (71.1, 89.2)
*pfdhps* A437G	104/130	80.0 (73.0, 85.5)	51/64	79.7 (70.4, 86.6)	53/66	80.3 (69.0, 88.2)
*pfdhps* K540E	4/130	3.08 (0.92, 9.75)	2/64	3.13 (0.43, 19.5)	2/66	3.03 (0.74, 11.5)
*pfdhfr + pfdhps-437*	91/130	70.0 (63.4, 75.8)	48/64	75.0 (66.3, 82.1)	43/66	65.2 (55.1, 74.0)
*pfdhfr* + *pfdhps*-437 + *pfdhps*-540	3/130	2.31 (0.75, 6.91)	1/64	1.56 (0.22, 10.4)	2/66	3.03 (0.74, 11.5)

Frequency of SP and AQ resistance mutations amongst study children with *P*. *falciparum* infection at the end-of-season cross-sectional surveys. When both wild-type and resistant mutants were present at a single codon, it was assumed that 2 clones were present, and both resistant mutants and wild-type clones were counted. The frequency of the mutation was then calculated as number of resistant mutants/total number of clones. Samples for which 2 or more codons had both mutant and wild type present were excluded. **Abbreviations:** AQ, amodiaquine; CI, confidence interval; *pfcrt*, *P*. *falciparum* chloroquine resistance transporter; *pfdhfr*, *P*. *falciparum* dihydrofolate reductase; *pfdhps*, *P*. *falciparum* dihydropteroate synthase; *pfmdr1*, *P*. *falciparum* multidrug resistance 1; SP, sulfadoxine-pyrimethamine.

The frequency of these resistance markers amongst school-age children not eligible for SMC but resident in the study area and thus exposed to the same circulating parasites was comparable with the frequency of mutations amongst parasites obtained from study children (Table 5). The combined *pfcrt* K76T + *pfmdr1* N86Y haplotype remained rare at the end of the study period, and the *pfdhps*-K540E mutation also remained at low frequency, with only 5 of 146 samples positive for this mutation in 2016.

**Table 5:** Frequency of molecular markers of SP and AQ resistance in schoolchildren—2014 and 2016.

	Burkina Faso		Mali	
Mutation	n/N	Frequency, % (95% CI)	n/N	Frequency, % (95% CI)
**2014**				
*pfcrt* K76T	9/66	13.6 (7.23, 24.2)	75/164	45.7 (38.2, 53.4)
*pfmdr1* N86Y	13/66	19.7 (11.8, 31.1)	9/165	5.45 (2.85, 10.2)
*pfcrt* K76T + *pfmdr1* N86Y	0/66	0	4/164	2.44 (0.91, 6.35)
*pfdhfr* C59R	74/79	93.7 (85.6, 97.4)	175/179	97.8 (94.2, 99.2)
*pfdhps* A437G	31/79	39.2 (29.1, 50.4)	72/179	40.2 (33.3, 47.6)
*pfdhps* K540E	2/79	2.53 (0.63, 9.62)	0/179	0
*pfdhfr* + *pfdhps*-437	30/79	38.0 (27.9, 49.1)	70/179	39.1 (32.2, 46.5)
*pfdhfr* + *pfdhps*-437 + *pfdhps*-540	1/79	1.27 (0.18, 8.51)	0/179	0
**2016**				
*pfcrt* K76T	7/61	11.5 (5.52, 22.3)	28/51	54.9 (41.1, 68.0)
*pfmdr1* N86Y	6/61	9.84 (4.45, 20.4)	6/52	11.5 (5.23, 23.6)
*pfcrt* K76T + *pfmdr1* N86Y	0/61	0	3/51	5.88 (1.89, 16.9)
*pfdhfr* C59R	68/73	93.2 (84.5, 97.1)	63/72	87.5 (77.6, 93.4)
*pfdhps* A437G	38/74	51.4 (40.0, 62.6)	27/70	38.6 (27.9, 50.5)
*pfdhps* K540E	3/74	4.05 (1.30, 11.9)	2/72	2.78 (0.69, 10.5)
*pfdhfr* + *pfdhps*-437	36/73	49.3 (38.0, 60.7)	26/70	37.1 (26.6, 49.1)
*pfdhfr* + *pfdhps*-437 + *pfdhps*-540	1/73	1.37 (0.19, 9.23)	1/70	1.43 (0.20, 9.60)

Frequency of SP and AQ resistance mutations amongst school-age children, resident in the study areas, with *P*. *falciparum* infection at the end-of-season surveys. When both wild-type and resistant mutants were present at a single codon, it was assumed that 2 clones were present, and both resistant mutants and wild-type clones were counted. The frequency of the mutation was then calculated as number of resistant mutants/total number of clones. Samples in which both mutant and wild type were detected at 2 or more codons were excluded. **Abbreviations:** AQ, amodiaquine; CI, confidence interval; *pfcrt*, *P*. *falciparum* chloroquine resistance transporter; *pfdhfr*, *P*. *falciparum* dihydrofolate reductase; *pfdhps*, *P*. *falciparum* dihydropteroate synthase; *pfmdr1*, *P*. *falciparum* multidrug resistance 1; SP, sulfadoxine-pyrimethamine.

### In vivo treatment efficacy of SP + AQ

Two hundred and eleven study children (58 in Burkina Faso, 153 in Mali) with asymptomatic *P*. *falciparum* infection at the final end-of-season survey in 2016 were recruited and treated with SP + AQ. Their age ranged from 7 to 63 months (including 4 children under 60 months in August 2016 and thus eligible for SMC who had since reached 5 years of age). Two children experienced early treatment failure, one in each country (S5 Table). In both cases, the child had a higher parasite density on day 2 than on day 0, although the densities were low in each case, and neither child was febrile (62 versus 48 parasites per microlitre in the child seen in Burkina Faso; 490 and 170 per microlitre in the child seen in Mali). There were no late parasitological failures (no asexual-stage parasitaemia) in Burkina Faso. There were 5 reinfections but no recrudescences in Mali. The unadjusted ACPR, including reinfections, was 98.3% (95% CI: 88.6%, 99.8%) in Burkina Faso and 96.1% (95% CI: 91.5%, 98.2%) in Mali. When reinfections were excluded, the ACPR rose to 99.3% (95% CI: 95.4%, 99.9%) in Mali.

## Discussion

Despite the very high level of malaria transmission in the 2 study districts, SMC appeared to provide a high level of protection against both malaria hospitalisations and deaths from malaria and uncomplicated clinical malaria; this was demonstrated by 3 key findings. Firstly, the incidence of malaria hospitalisations and deaths from malaria and episodes of uncomplicated clinical malaria was markedly lower during the SMC period (August to November) than in the month immediately preceding it, with evidence that children who received SMC in a particular month were very well protected. Secondly, incidence of malaria hospitalisations and deaths from malaria and clinical malaria was substantially lower during the first 4 weeks after an SMC course than in subsequent time periods. The rate ratios obtained and the profile of protection over time were compatible with results in earlier trials and with observational studies [25]. Thirdly, the prevalence of *P*. *falciparum* was markedly lower in children with recent SMC at both the end-of-season surveys and the weekly surveys carried out during the rainy season. The remaining burden amongst study children therefore reflects the exceptionally high infection rates to which study children were exposed, illustrated by a prevalence of *P*. *falciparum* parasitaemia in excess of 50% amongst school-age children resident in the same areas.

The observed high level of protection from SMC against malaria is compatible with the current low prevalence of key molecular markers of SP and AQ resistance observed in the cross-sectional surveys, the very high curative efficacy of the SMC drug combination in treatment, and results from other studies that have evaluated SMC in the Sahel region during the period of the trial [15]. A series of case–control studies conducted as part of the ACCESS-SMC programme in 5 countries, including Burkina Faso and Mali, compared children with and without recent SMC (i.e., similar to the comparisons done prospectively in the present analysis). The case–control approach found evidence of a high level of protection for the first 28 days after administration [16], comparable with that seen here, and with previous SMC trials [1,2]. Taken together, these results suggest that SMC should continue to be a priority intervention for malaria control in areas of intense seasonal transmission. Implementation should be supported in appropriate epidemiological situations in which it is not yet deployed, and coverage should be maximised when SMC is already in place. The results from children prior to receipt of SMC or who missed SMC during the intervention period indicate that there could be a major increase in burden in the areas identified as a current priority by WHO [12] if SMC programmes are not supported. The high incidence in children who miss SMC in a particular month underscores the importance of coverage of all monthly courses for full protection and the value of this as a target for programme evaluation [26].

Strengths of this study include the large sample size, the careful documentation of all SMC courses (and daily doses) and morbidity episodes, and the use of tablet PCs (specifically, the use of QR codes and time/date stamps) to link all contacts to the correct child with a very high degree of accuracy. Because the main trial was powered to investigate hospital admissions and mortality as a primary outcome, these analyses have high statistical power to investigate uncomplicated malaria and severe malaria by time since SMC in more detail than has been possible in previous (smaller) trials.

However, there are a number of limitations to this study. Given the large scale of the study and the need to manage children who were unwell promptly, it was not possible for blood smears to be prepared for all suspected malaria cases. The subset of children for which both an RDT and blood smear were available indicate that around a third of children positive by RDT may no longer carry parasites; this was similar in both countries. Low specificity is likely to be a consequence of parasite antigens remaining in the circulation of children whose infection has been cleared by SMC or treatment, as observed in a previous SMC study in Senegal [6]. Overdiagnosis is likely to be a feature of most programmatic data because diagnosis of malaria in the routine system relies on the use of RDTs. However, even if the burden observed in this study were to be discounted to account for potential overdiagnosis, it would remain substantial.

Deaths that occurred in the community were diagnosed by verbal autopsy, and thus parasitological confirmation of malaria was not usually possible. Comparison of the intervention effect over the study period is made difficult by the variability in transmission intensity each year in these and similar study areas and by the differences in the timing of SMC and end-of-season surveys (almost a month later in 2014 than in 2015 and 2016). In the analyses of malaria incidence, children with and without recent SMC were compared, but since children without SMC in a particular month were those who missed their scheduled SMC, this was not a randomly allocated exposure. It is possible that children with poor SMC coverage live in more remote parts of the community and have higher malaria risk. When comparing children on the basis of their adherence to SMC, the analysis strategy attempted to minimise confounding through this mechanism by including only those children who received at least 3 of the 4 scheduled SMC courses in that calendar year (i.e., children who missed a particular SMC course received all other courses that year).

Prevalence ratios were calculated as a simple way to show the large differences in carriage of parasites between those with and without SMC. However, the specific values of the prevalence ratios may not be widely generalisable (e.g., the prevalence ratio of 11.8 observed in 2014 in Burkina Faso could not apply in areas with prevalence amongst SMC recipients in excess of 8.5% because prevalence is constrained between 0% and 100%).

Although the prevalence of resistance markers was low in 2014 and in 2016, it will be important to monitor longer-term changes on molecular markers of resistance. The timing of the treatment efficacy study towards the end of the malaria transmission season is likely to have limited the number of reinfections to which study children were exposed. It is also unclear whether curative efficacy would differ in children with higher-density infections, although these children would be more likely to have symptoms and thus be treated with artemether-lumefantrine, the first-line treatment regimen in both study areas, rather than SMC.

Despite the positive findings with respect to the benefit of SMC in the study areas, these results show that more must be done to protect young children from the consequences of malaria in areas of intense seasonal transmission. It is clear that missed SMC courses result in a markedly elevated risk of both uncomplicated and malaria hospitalisations and deaths from malaria, and thus, it is important to ensure that delivery reaches as many children as possible each month. This may require a mixture of delivery strategies (e.g., door-to-door delivery with fixed points available if these contacts are missed).

Our findings indicate that 4 courses of SMC are not sufficient to cover the entire malaria transmission season in the southern part of Burkina Faso and Mali, where rainfall peaks over a longer period than in the northern parts of the Sahel region. In each year of the study, a large number of cases of uncomplicated malaria, malaria hospitalisation, and deaths from malaria were recorded in July, prior to SMC delivery in August. Providing additional SMC courses may, therefore, have an important impact on the malaria burden. SMC programmes using 5 or 6 courses should be evaluated in these areas and other areas at similar latitudes, such as northern Ghana, that have similar epidemiology [27,28] and large populations at risk. Additional SMC courses could help mitigate against the risks of introducing drug resistance by helping to avoid situations in which either SMC begins too late, and thus SP and AQ are used to clear high-density infections from a large proportion of children [29], or SMC finishes too soon, leaving children exposed to reinfection when the residual concentrations of SMC drugs have fallen below therapeutic levels.

Additional interventions are also needed to reduce the malaria risk when SMC courses are inevitably missed. Seasonal vaccination with a malaria vaccine [14] may be one approach, and the RTS,S/AS01 malaria vaccine is currently being evaluated as an addition to SMC in the study districts (clinicaltrials.gov NCT03143218). However, unless these approaches are strongly synergistic, it is likely that even further interventions will be needed, such as additional methods of vector control, for these areas where malaria has proved most challenging to control and continues to overload the health system.

In summary, SMC remains highly protective in the trial areas, and our results emphasise the need for continued support of SMC programmes. However, the high incidence of malaria in July each year emphasises the need for a fifth monthly course of SMC to adequately cover the whole transmission season. Additional measures beyond SMC and LLINs are needed to further reduce malaria burden in these areas and areas with similar epidemiology.

## Supporting information

S1 Methods Additional detail on laboratory methods. (DOCX)

S1 Fig Location of the study districts within Burkina Faso and Mali. Base map adapted from digitised boundaries of health districts in Burkina Faso and Mali. Noor AM, Kibuchi E, Mitto B, Coulibaly D, Doumbo OK, Snow RW. Sub-National Targeting of Seasonal Malaria Chemoprevention in the Sahelian Countries of the Nouakchott Initiative. PLoS One. 2015;10(8):e0136919. https://doi.org/10.1371/journal.pone.0136919.s001.
(DOCX)

S2 Fig Location of the study health centres and the hospital within Houndé District, Burkina Faso. Data points from study GPS. Study health centres are shown by blue circles. The study hospital is shown by a red square. Base map adapted from digitised boundaries of health districts in Burkina Faso. Noor AM, Kibuchi E, Mitto B, Coulibaly D, Doumbo OK, Snow RW. Sub-National Targeting of Seasonal Malaria Chemoprevention in the Sahelian Countries of the Nouakchott Initiative. PLoS One. 2015;10(8):e0136919. https://doi.org/10.1371/journal.pone.0136919.s001. GPS, Global Positioning System.
(DOCX)

S3 Fig Location of the study health centres and the hospital within Bougouni District, Mali. Data points from study GPS. Study health centres are shown by blue circles. The study hospital is shown by a red square. Base map adapted from digitised boundaries of health districts in Mali. Noor AM, Kibuchi E, Mitto B, Coulibaly D, Doumbo OK, Snow RW. Sub-National Targeting of Seasonal Malaria Chemoprevention in the Sahelian Countries of the Nouakchott Initiative. PLoS One. 2015;10(8):e0136919. https://doi.org/10.1371/journal.pone.0136919.s001. GPS, Global Positioning System.
(DOCX)

S4 Fig Incidence of deaths from malaria and nonfatal hospital admissions by age group and country. Incidence of malaria hospitalisations and deaths from malaria (left panel) and clinical malaria (right panel) by age group over the study period. Malaria hospitalisations and deaths from malaria were defined as hospital admission with a diagnosis of malaria and blood-slide–or RDT-confirmed *P*. *falciparum* infection or deaths for which malaria was listed as the primary diagnosis. Clinical malaria was defined as attendance at study health facility with a history of fever or measured temperature ≥37.5 °C, with malaria infection confirmed by RDT. Incidence rates are presented per 1,000 person-years and include repeat events in the same child, provided the healthcare contact occurred more than 7 days apart. Vertical bars show 95% CIs. CI, confidence interval; RDT, rapid diagnostic test.
(DOCX)

S5 Fig Incidence of malaria hospitalisations and deaths from malaria and incidence of episodes of uncomplicated malaria during the study period in the SMC + placebo group—Burkina Faso. Incidence of malaria hospitalisations and deaths from malaria (a) and clinical malaria (b) by calendar month over the study period in Houndé District, Burkina Faso. (c) shows the incidence of uncomplicated malaria during the period when SMC was delivered (shown by vertical dashed lines) amongst children who had received SMC within the previous 28 days or who had not received recent SMC (no SMC in the previous 35 days). The analysis of children with ‘no recent SMC’ was restricted to children who received 3 courses of SMC during that intervention year (i.e., this excludes children who missed SMC on more than one occasion). Malaria hospitalisations and deaths from malaria were defined as hospital admission with a diagnosis of malaria and blood-slide–or RDT-confirmed *P*. *falciparum* infection or deaths for which malaria was listed as the primary diagnosis. Clinical malaria was defined as attendance at study health facility with a history of fever or measured temperature ≥37.5 °C, with malaria infection confirmed by RDT. Incidence rates are presented per 1,000 person-months at risk rather than per 1,000 person-years and include repeat events in the same child, provided the healthcare contact occurred more than 7 days apart. Vertical bars show 95% CIs. Note that in Burkina Faso, in 2015, one-third of the study cohort received SMC 1 month late because of the global shortage of SP-AQ in 2015. AQ, amodiaquine; CI, confidence interval; RDT, rapid diagnostic test; SMC, seasonal malaria chemoprevention; SP, sulfadoxine-pyrimethamine.
(DOCX)

S6 Fig Incidence of malaria hospitalisations and deaths from malaria and incidence of episodes of uncomplicated malaria during the study period in the SMC + placebo group—Mali. Incidence of malaria hospitalisations and deaths from malaria (a) and clinical malaria (b) by calendar month over the study period in Bougouni District, Mali. (c) shows the incidence of uncomplicated malaria during the period when SMC was delivered (shown by vertical dashed lines) amongst children who had received SMC within the previous 28 days or who had not received recent SMC (no SMC in the previous 35 days). The analysis of children with ‘no recent SMC’ was restricted to children who received 3 courses of SMC during that intervention year (i.e., this excludes children who missed SMC on more than one occasion). Malaria hospitalisations and deaths from malaria were defined as hospital admission with a diagnosis of malaria and blood-slide–or RDT-confirmed *P*. *falciparum* infection or deaths for which malaria was listed as the primary diagnosis. Clinical malaria was defined as attendance at study health facility with a history of fever or measured temperature ≥37.5 °C, with malaria infection confirmed by RDT. Incidence rates are presented per 1,000 person-months at risk rather than per 1,000 person-years and include repeat events in the same child, provided the healthcare contact occurred more than 7 days apart. Vertical bars show 95% CIs. CI, confidence interval; RDT, rapid diagnostic test; SMC, seasonal malaria chemoprevention.
(DOCX)

S1 Table Number of cycles received per year by each study child in the SMC plus placebo group. SMC, seasonal malaria chemoprevention.
(DOCX)

S2 Table Number of children receiving all 3 daily doses of SMC amongst those who received the first dose of each monthly course. SMC, seasonal malaria chemoprevention.
(DOCX)

S3 Table Prevalence of malaria parasitaemia at the end-of-season surveys amongst children in the SMC + placebo group comparing children with recent SMC and those who missed the last SMC cycle but who otherwise received all SMC courses that year. Results from both countries are combined. SMC, seasonal malaria chemoprevention.
(DOCX)

S4 Table Prevalence of molecular markers of resistance to AQ and SP amongst children in the SMC + placebo group with parasitaemia overall and by study country. Prevalence of SP and AQ resistance mutations amongst study children with *P*. *falciparum* infection at the end-of-season surveys in both study centres overall, in Houndé District, Burkina Faso and in Bougouni District, Mali. Prevalence is calculated as number of resistant mutations/number of children, with mixed mutations at a single locus counted amongst those resistant. Samples in which both mutant and wild type were detected at 2 or more codons were excluded. AQ, amodiaquine; SMC, seasonal malaria chemoprevention; SP, sulfadoxine-pyrimethamine.
(DOCX)

S5 Table Results of 28-day treatment efficacy protocol. ACPR, adequate clinical and parasitological response; PCR, polymerase chain reaction.
(DOCX)

S1 STROBE Checklist STROBE checklist. STROBE, Strengthening the Reporting of Observational Studies in Epidemiology.
(DOCX)

## Abbreviations

ACPR: adequate clinical and parasitological response
ACT: artemisinin-based combination therapy
AQ: amodiaquine
AZ: azithromycin
CI: confidence interval
HRP2: histidine-rich protein 2
LLIN: long-lasting insecticide-treated net
*pfcrt*: *Plasmodium falciparum* chloroquine resistance transporter
*pfdhfr*: *Plasmodium falciparum* dihydrofolate reductase
*pfdhps*: *Plasmodium falciparum* dihydropteroate synthase
*pfmdr1*: *Plasmodium falciparum* multidrug resistance 1
QR: Quick Response
RDT: rapid diagnostic test
SMC: seasonal malaria chemoprevention
SP: sulfadoxine-pyrimethamine
