# Supplementary material for: Evaluation of seasonal malaria chemoprevention in two areas of intense seasonal malaria transmission: Secondary analysis of a household-randomised, placebo-controlled trial in Houndé District, Burkina Faso and Bougouni District, Mali
Source: PLoS Med. 2020 Aug 21;17(8):e1003214. doi: 10.1371/journal.pmed.1003214 (PMC7442230; doi:10.1371/journal.pmed.1003214)
Supplement: S1 Methods — (DOCX) [file pmed.1003214.s001.docx]

**S1 Methods:**

**Measurement of the frequency and prevalence of molecular markers of SP and AQ resistance**

The presence of molecular markers of resistance to SP and AQ was determined among study children with *P. falciparum* infection, and from approximately 50 school-age children who tested positive for malaria, at the end of each transmission season.

DNA was extracted from filter paper strips as described previously (Plowe et. al, 1995)^1^. Briefly, an approximately 2× 5 mm piece of blood-soaked filter paper was placed in 50 μl of methanol for 15 min. The methanol was poured off and the paper heated at 95-100°C in 50 μl of water for 10 minutes. Five microliters of the resulting solution was used as a PCR template for the first round of a Nested PCR. One microliter of the product of the first PCR was amplified in the second round of PCR. Samples that failed to yield PCR amplification with the methanol method were re-extracted using an alternative chelex-based method. Dhfr mutations at codon 59, dhps mutations at codons 437 and 540, P. falciparum chloroquine transporter gene (pfcrt) mutations at codon 76 and P. falciparum multidrug resistance gene one (pfmdr1)at codon 86 were analysed by nested polymerase chain reaction (PCR) and/or PCR-restriction fragment length polymorphism (RFLP)according to published methods ^1,2^.

**Calculation of frequency and prevalence of resistance markers**

Where both wild type and resistant mutants were present at a single codon, it was assumed that two clones were present, and both resistant mutants and wild-type clones were counted. The frequency of the mutation was calculated as the number of resistant mutants / the total number of clones. The prevalence of mutations among study children was calculated as the number of resistant mutants / the number of study children, with infections carrying mixed mutations at a single locus counted among those resistant. Samples in which both mutant and wild type were detected at two or more codons were excluded from the calculation of both frequency and prevalence.

References:

1. Plowe, C. V., Djimde, A., Bouare, M., Doumbo, O. & Wellems, T. E. Pyrimethamine and proguanil resistance-conferring mutations in Plasmodium falciparum dihydrofolate reductase: polymerase chain reaction methods for surveillance in Africa. Am J Trop Med Hyg 52, 565-568, doi:10.4269/ajtmh.1995.52.565 (1995).

2. Djimde, A. et al. A molecular marker for chloroquine-resistant falciparum malaria. N Engl J Med 344, 257-263, doi:10.1056/nejm200101253440403 (2001).
