# Supplementary material for: Evaluation of seasonal malaria chemoprevention in two areas of intense seasonal malaria transmission: Secondary analysis of a household-randomised, placebo-controlled trial in Houndé District, Burkina Faso and Bougouni District, Mali
Source: PLoS Med. 2020 Aug 21;17(8):e1003214. doi: 10.1371/journal.pmed.1003214 (PMC7442230; doi:10.1371/journal.pmed.1003214)
Supplement: S2 Fig — Data points from study GPS. Study health centres are shown by blue circles. The study hospital is shown by a red square. Base map adapted from digitised boundaries of health districts in Burkina Faso. Noor AM, Kibuchi E, Mitto B, Coulibaly D, Doumbo OK, Snow RW. Sub-National Targeting of Seasonal Malaria Chemoprevention in the Sahelian Countries of the Nouakchott Initiative. PLoS One. 2015;10(8):e0136919. https://doi.org/10.1371/journal.pone.0136919.s001. GPS, Global Positioning System. (DOCX) [file pmed.1003214.s003.docx]

**S2 Fig. Location of the study health centres and the hospital within Houndé District, Burkina Faso**


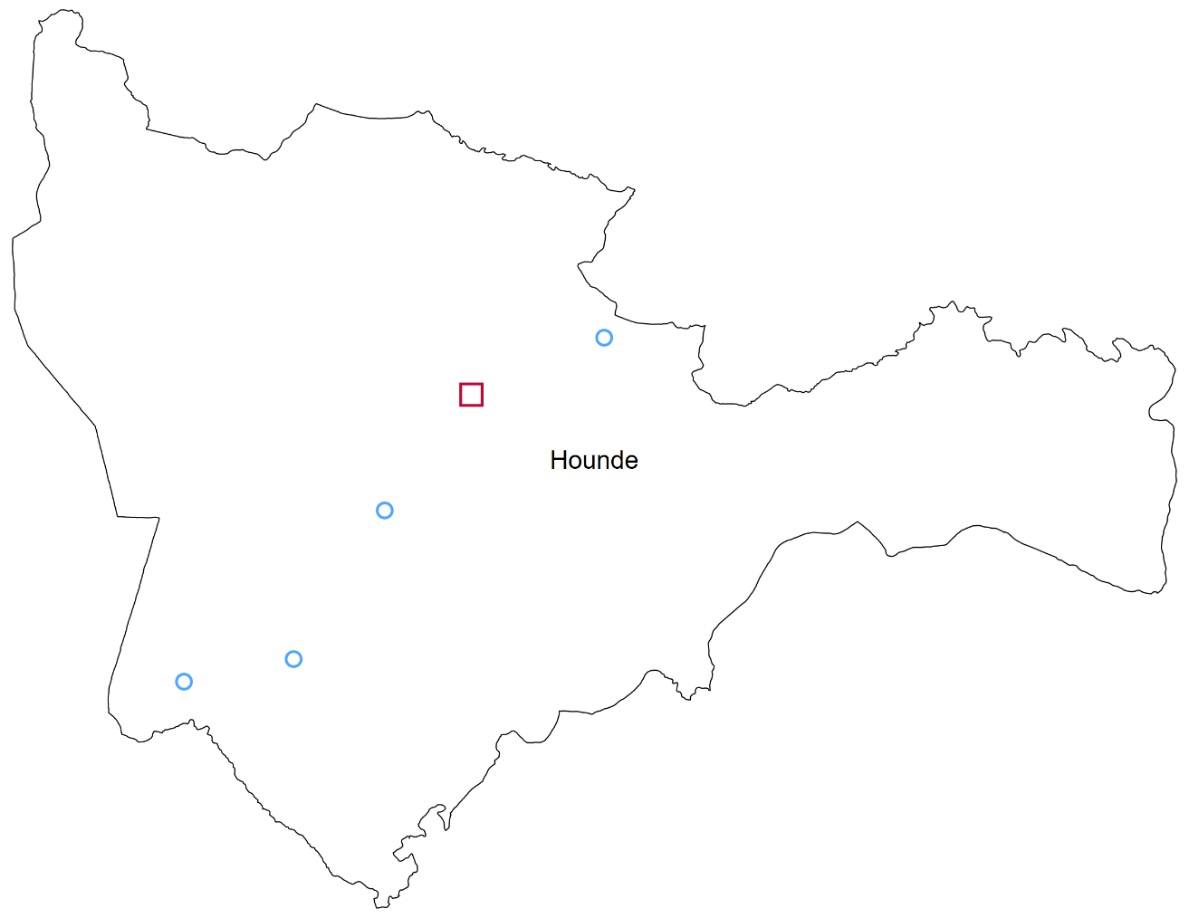


Data points from study GPS. Study health centres are shown by blue circles. The study hospital is shown by a red square. Base map adapted from digitised boundaries of health districts in Burkina Faso. Noor et al. Sub-National Targeting of Seasonal Malaria Chemoprevention in the Sahelian Countries of the Nouakchott Initiative. PLoS One. 2015;10(8):e0136919. <https://doi.org/10.1371/journal.pone.0136919.s001>
