# Supplementary material for: Evaluation of seasonal malaria chemoprevention in two areas of intense seasonal malaria transmission: Secondary analysis of a household-randomised, placebo-controlled trial in Houndé District, Burkina Faso and Bougouni District, Mali
Source: PLoS Med. 2020 Aug 21;17(8):e1003214. doi: 10.1371/journal.pmed.1003214 (PMC7442230; doi:10.1371/journal.pmed.1003214)
Supplement: S4 Fig — Incidence of malaria hospitalisations and deaths from malaria (left panel) and clinical malaria (right panel) by age group over the study period. Malaria hospitalisations and deaths from malaria were defined as hospital admission with a diagnosis of malaria and blood-slide–or RDT-confirmed P. falciparum infection or deaths for which malaria was listed as the primary diagnosis. Clinical malaria was defined as attendance at study health facility with a history of fever or measured temperature ≥37.5 °C, with malaria infection confirmed by RDT. Incidence rates are presented per 1,000 person-years and include repeat events in the same child, provided the healthcare contact occurred more than 7 days apart. Vertical bars show 95% CIs. CI, confidence interval; RDT, rapid diagnostic test. (DOCX) [file pmed.1003214.s005.docx]

**S4 Fig. Incidence of deaths from malaria, and non-fatal hospital admissions by age group and country.**

**
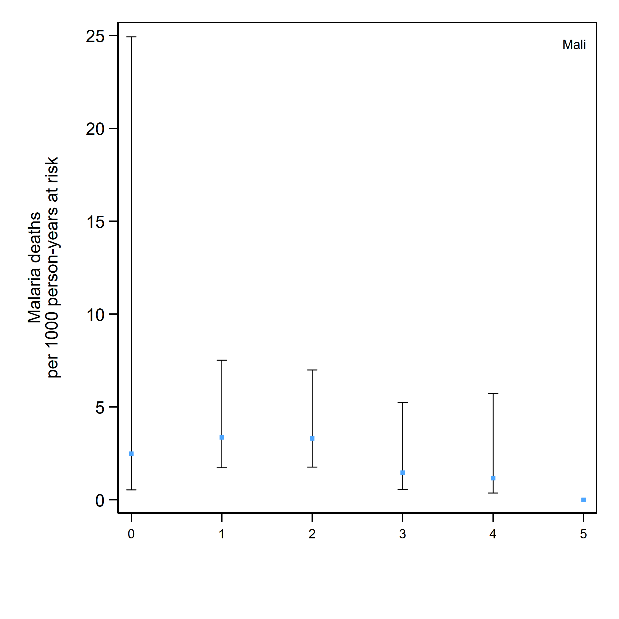

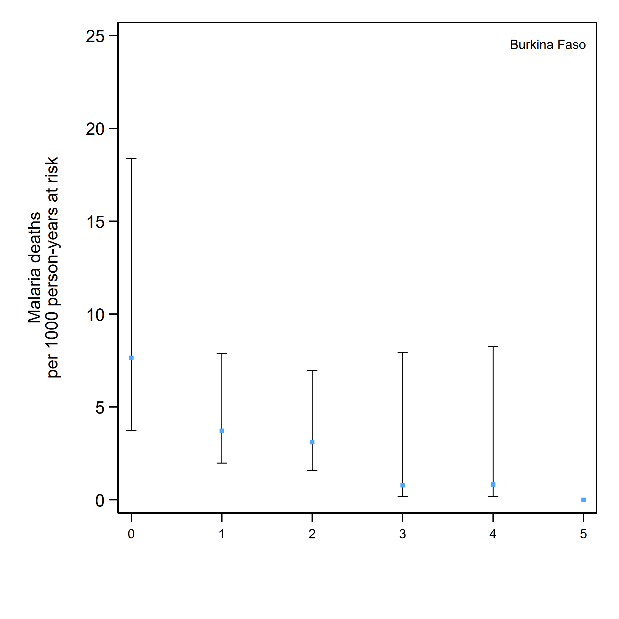

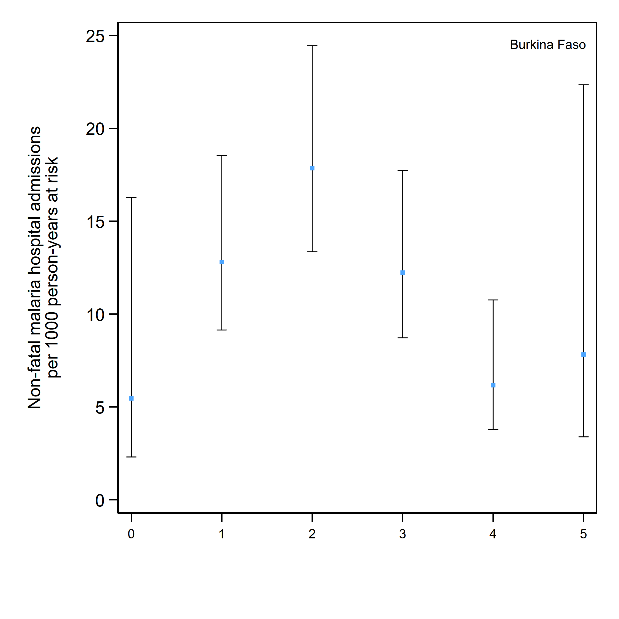

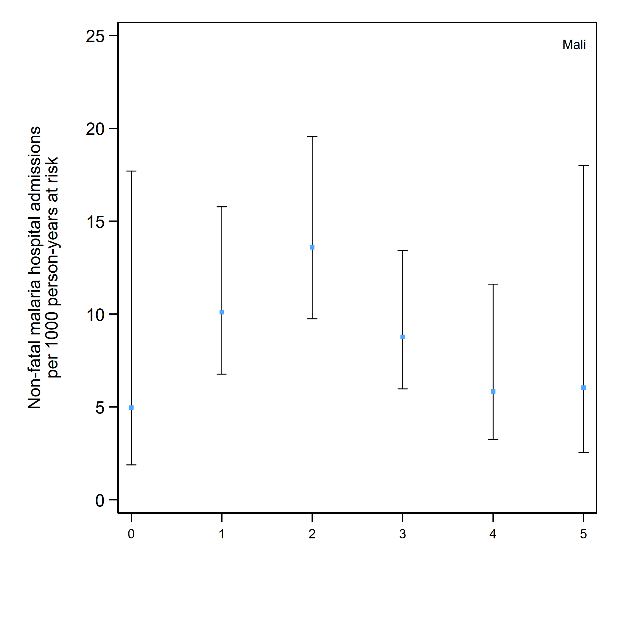
**

**Figure legend:** Incidence of malaria hospitalisations and deaths from malaria (left panel) and clinical malaria (right panel) by age group over the study period. Malaria hospitalisations and deaths from malaria were defined as hospital admission with a diagnosis of malaria and blood-slide or RDT-confirmed *P. falciparum* infection, or deaths for which malaria was listed as the primary diagnosis. Clinical malaria was defined as attendance at study health facility with a history of fever or measured temperature >=37.5° C, with malaria infection confirmed by rapid diagnostic test. Incidence rates are presented per 1000 person-years, and include repeat events in the same child, provided the healthcare contact occurred more than 7 days apart. Vertical bars show 95% confidence intervals.
