# Supplementary material for: Evaluation of seasonal malaria chemoprevention in two areas of intense seasonal malaria transmission: Secondary analysis of a household-randomised, placebo-controlled trial in Houndé District, Burkina Faso and Bougouni District, Mali
Source: PLoS Med. 2020 Aug 21;17(8):e1003214. doi: 10.1371/journal.pmed.1003214 (PMC7442230; doi:10.1371/journal.pmed.1003214)
Supplement: S5 Fig — Incidence of malaria hospitalisations and deaths from malaria (a) and clinical malaria (b) by calendar month over the study period in Houndé District, Burkina Faso. (c) shows the incidence of uncomplicated malaria during the period when SMC was delivered (shown by vertical dashed lines) amongst children who had received SMC within the previous 28 days or who had not received recent SMC (no SMC in the previous 35 days). The analysis of children with ‘no recent SMC’ was restricted to children who received 3 courses of SMC during that intervention year (i.e., this excludes children who missed SMC on more than one occasion). Malaria hospitalisations and deaths from malaria were defined as hospital admission with a diagnosis of malaria and blood-slide–or RDT-confirmed P. falciparum infection or deaths for which malaria was listed as the primary diagnosis. Clinical malaria was defined as attendance at study health facility with a history of fever or measured temperature ≥37.5 °C, with malaria infection confirmed by RDT. Incidence rates are presented per 1,000 person-months at risk rather than per 1,000 person-years and include repeat events in the same child, provided the healthcare contact occurred more than 7 days apart. Vertical bars show 95% CIs. Note that in Burkina Faso, in 2015, one-third of the study cohort received SMC 1 month late because of the global shortage of SP-AQ in 2015. AQ, amodiaquine; CI, confidence interval; RDT, rapid diagnostic test; SMC, seasonal malaria chemoprevention; SP, sulfadoxine-pyrimethamine. (DOCX) [file pmed.1003214.s006.docx]

**S5 Fig. Incidence of malaria hospitalisations and deaths from malaria and incidence of episodes of uncomplicated malaria during the study period in the SMC + placebo group – Burkina Faso**


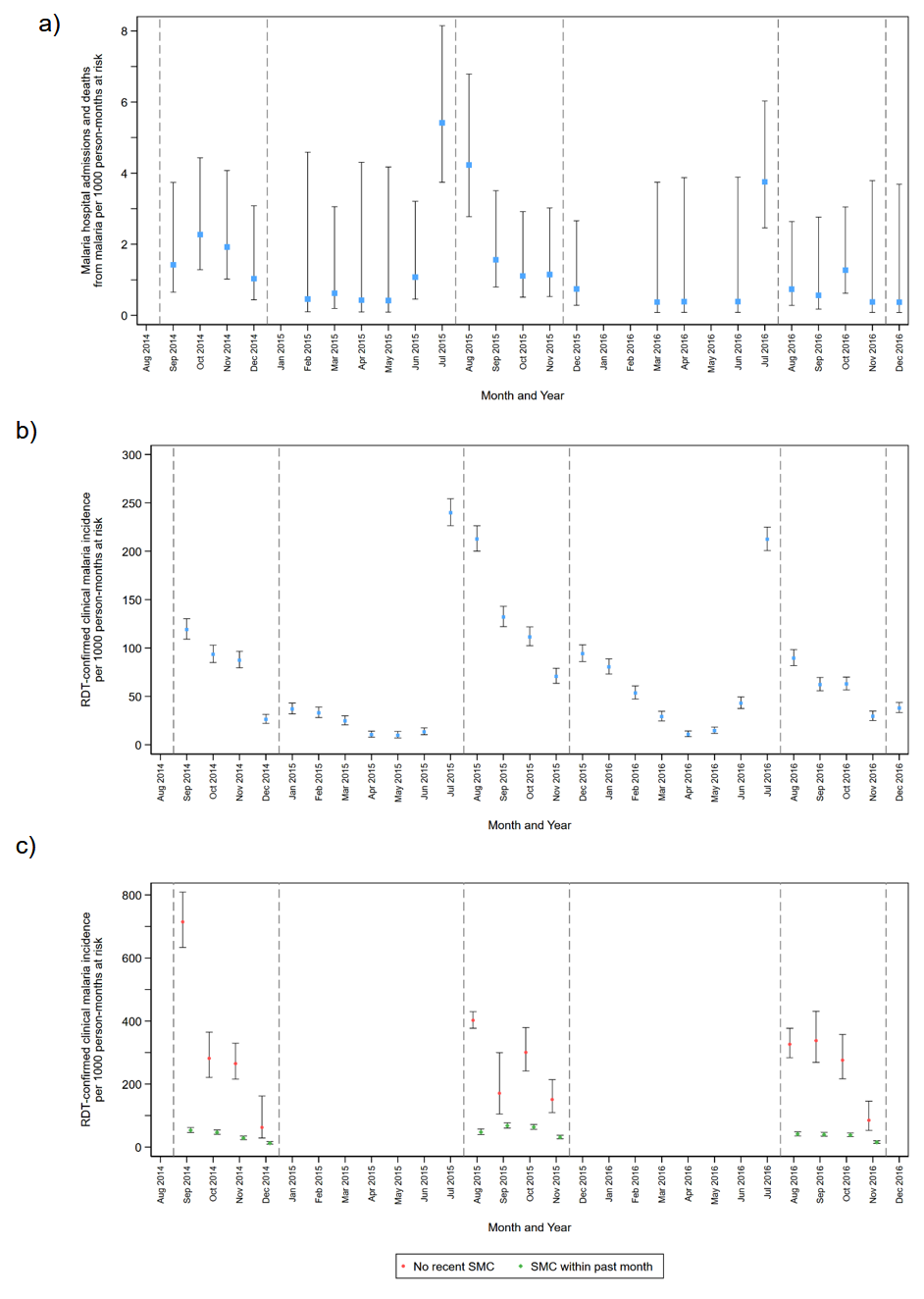


**Figure legend**: Incidence of malaria hospitalisations and deaths from malaria (Panel a), and clinical malaria (Panel b) by calendar month over the study period in Houndé District, Burkina Faso. Panel c) shows the incidence of uncomplicated malaria during the period when SMC was delivered (shown by vertical dashed lines), among children who had received SMC within the previous 28 days, or who had not received recent SMC (no SMC in the previous 35 days). The analysis of children with ‘no recent SMC’ was restricted to children who received three courses of SMC during that intervention year (i.e. this excludes children who missed SMC on more than one occasion). Malaria hospitalisations and deaths from malaria were defined as hospital admission with a diagnosis of malaria and blood slide- or RDT-confirmed *P. falciparum* infection, or deaths for which malaria was listed as the primary diagnosis. Clinical malaria was defined as attendance at study health facility with a history of fever or measured temperature >=37.5° C, with malaria infection confirmed by rapid diagnostic test. Incidence rates are presented per 1000 person-months at risk, rather than per 1000 person-years, and include repeat events in the same child, provided the healthcare contact occurred more than 7 days apart. Vertical bars show 95% confidence intervals. Note that in Burkina Faso, in 2015, one third of the study cohort received SMC one month late, due to the global shortage of SP-AQ in 2015.
