# Supplementary material for: Evaluation of seasonal malaria chemoprevention in two areas of intense seasonal malaria transmission: Secondary analysis of a household-randomised, placebo-controlled trial in Houndé District, Burkina Faso and Bougouni District, Mali
Source: PLoS Med. 2020 Aug 21;17(8):e1003214. doi: 10.1371/journal.pmed.1003214 (PMC7442230; doi:10.1371/journal.pmed.1003214)
Supplement: S1 Table — SMC, seasonal malaria chemoprevention. (DOCX) [file pmed.1003214.s008.docx]

**S1 Table.** Number of cycles received per year by each study child in the SMC plus placebo group

| **Number of cycles received** | **Burkina Faso** | | **Mali** | | | **Both Centres combined** | | |
| --- | --- | --- | --- | --- | --- | --- | --- | --- |
| **2014** | **No. children** | **%** | **No. children** | **%** | **No. children** | | **%** |  |
| 0 | 24 | 0.5 | 43 | 0.89 | 67 | | 0.70 |  |
| 1 | 64 | 1.35 | 124 | 2.56 | 188 | | 1.96 |  |
| 2 | 208 | 4.37 | 263 | 5.43 | 471 | | 4.90 |  |
| 3 | 877 | 18.4 | 817 | 16.9 | 1694 | | 17.6 |  |
| 4 | 3585 | 75.4 | 3598 | 74.3 | 7183 | | 74.8 |  |
| Total | 4758 |  | 4845 |  | 9603 | |  |  |
| **2015** |  |  |  |  | **No. children** | | **%** |  |
| 0 | 333 | 6.25 | 266 | 4.89 | 599 | | 5.57 |  |
| 1 | 66 | 1.24 | 118 | 2.17 | 184 | | 1.71 |  |
| 2 | 346 | 6.50 | 328 | 6.03 | 674 | | 6.26 |  |
| 3 | 1122 | 21.1 | 1137 | 20.9 | 2259 | | 21.0 |  |
| 4 | 3457 | 64.9 | 3587 | 66.0 | 7044 | | 65.5 |  |
| Total | 5324 |  | 5436 |  | 10760 | |  |  |
| **2016** |  |  |  |  | **No. children** | | **%** |  |
| 0 | 425 | 7.84 | 444 | 7.89 | 869 | | 7.87 |  |
| 1 | 76 | 1.40 | 143 | 2.54 | 219 | | 1.98 |  |
| 2 | 233 | 4.30 | 407 | 7.24 | 640 | | 5.79 |  |
| 3 | 977 | 18.0 | 1165 | 20.7 | 2142 | | 19.4 |  |
| 4 | 3712 | 68.5 | 3465 | 61.6 | 7177 | | 65.0 |  |
| Total | 5423 |  | 5624 |  | 11047 | |  |  |
