# Supplementary material for: Evaluation of seasonal malaria chemoprevention in two areas of intense seasonal malaria transmission: Secondary analysis of a household-randomised, placebo-controlled trial in Houndé District, Burkina Faso and Bougouni District, Mali
Source: PLoS Med. 2020 Aug 21;17(8):e1003214. doi: 10.1371/journal.pmed.1003214 (PMC7442230; doi:10.1371/journal.pmed.1003214)
Supplement: S2 Table — SMC, seasonal malaria chemoprevention. (DOCX) [file pmed.1003214.s009.docx]

**S2 Table**. Number of children receiving all 3 daily doses of SMC, among those who received the first dose of each monthly course.

| Year | SMC course | Burkina Faso |  |  | Mali |
| --- | --- | --- | --- | --- | --- |
|  |  | n/N | % | n/N | % |
| 2014 | 1 | 4051/4279 | 94.7 | 3955/4336 | 91.2 |
|  | 2 | 4205/4344 | 96.8 | 4023/4308 | 93.4 |
|  | 3 | 4205/4315 | 97.5 | 4143/4394 | 94.3 |
|  | 4 | 4429/4513 | 98.1 | 4287/4455 | 96.2 |
|  |  |  |  |  |  |
| 2015 | 1 | 4350/4394 | 99.0 | 4424/4599 | 96.2 |
|  | 2 | 4515/4546 | 99.3 | 4631/4734 | 97.8 |
|  | 3 | 4473/4505 | 99.3 | 4592/4691 | 97.9 |
|  | 4 | 4488/4507 | 99.6 | 4471/4509 | 99.2 |
|  |  |  |  |  |  |
| 2016 | 1 | 4443/4478 | 99.2 | 4334/4478 | 96.8 |
|  | 2 | 4591/4606 | 99.7 | 4727/4762 | 99.3 |
|  | 3 | 4598/4608 | 99.8 | 4439/4484 | 99.0 |
|  | 4 | 4622/4629 | 99.9 | 4547/4588 | 99.1 |
