# Supplementary material for: Evaluation of seasonal malaria chemoprevention in two areas of intense seasonal malaria transmission: Secondary analysis of a household-randomised, placebo-controlled trial in Houndé District, Burkina Faso and Bougouni District, Mali
Source: PLoS Med. 2020 Aug 21;17(8):e1003214. doi: 10.1371/journal.pmed.1003214 (PMC7442230; doi:10.1371/journal.pmed.1003214)
Supplement: S3 Table — Results from both countries are combined. SMC, seasonal malaria chemoprevention. (DOCX) [file pmed.1003214.s010.docx]

**S3 Table.** Prevalence of malaria parasitaemia at the end of season surveys among children in the SMC + placebo group, comparing children with recent SMC, and those who missed the last SMC cycle but who otherwise received all SMC courses that year.

|  | **Children who received the final SMC cycle before the survey** | | **Children missed the final SMC cycle, but received the 3 earlier SMC cycles** | |  |  |
| --- | --- | --- | --- | --- | --- | --- |
| **Year** | **n/N** | **Prevalence**  **(95% CI)** | **n/N** | **Prevalence**  **(95% CI)** | **Prevalence Ratio**  **(95% CI)** | **P-value** |
| 2014 | 69/1926 | 3.58 (2.83, 4.52) | 7/30 | 23.3 (11.5, 41.5) | 6.51 (3.27, 13.0) | <0.001 |
| 2015 | 124/1781 | 6.96 (5.87, 8.25) | 26/93 | 28.0 (19.2, 38.8) | 4.01 (2.71, 5.94) | <0.001 |
| 2016 | 131/1815 | 7.22 (6.09, 8.54) | 18/80 | 22.5 (14.6, 33.0) | 3.12 (2.02, 4.82) | <0.001 |

**Table legend:** Results from both countries are combined.
