# Supplementary material for: Evaluation of seasonal malaria chemoprevention in two areas of intense seasonal malaria transmission: Secondary analysis of a household-randomised, placebo-controlled trial in Houndé District, Burkina Faso and Bougouni District, Mali
Source: PLoS Med. 2020 Aug 21;17(8):e1003214. doi: 10.1371/journal.pmed.1003214 (PMC7442230; doi:10.1371/journal.pmed.1003214)
Supplement: S4 Table — Prevalence of SP and AQ resistance mutations amongst study children with P. falciparum infection at the end-of-season surveys in both study centres overall, in Houndé District, Burkina Faso and in Bougouni District, Mali. Prevalence is calculated as number of resistant mutations/number of children, with mixed mutations at a single locus counted amongst those resistant. Samples in which both mutant and wild type were detected at 2 or more codons were excluded. AQ, amodiaquine; SMC, seasonal malaria chemoprevention; SP, sulfadoxine-pyrimethamine. (DOCX) [file pmed.1003214.s011.docx]

**S4 Table.** Prevalence of molecular markers of resistance to AQ and SP among children in the SMC + placebo group with parasitaemia, overall and by study country

|  | **Overall** | | **Burkina Faso** | | **Mali** | |
| --- | --- | --- | --- | --- | --- | --- |
| **Mutation** | n/N | Prevalence, %  (95% CI) | n/N | Prevalence, %  (95% CI) | n/N | Prevalence, %  (95% CI) |
| **2014** |  |  |  |  |  |  |
| *pfcrt* K76T | 59/83 | 71.1 (60.3, 79.9) | 14/27 | 51.9 (33.0, 70.2) | 45/56 | 80.4 (67.7, 88.9) |
| *pfmdr1* N86Y | 22/83 | 26.5 (18.2, 37.0) | 7/27 | 25.9 (12.5, 46.1) | 15/56 | 26.8 (16.9, 39.7) |
| *pfcrt K76T + pfmdr1* N86Y | 17/83 | 20.5 (13.2, 30.4) | 4/27 | 14.8 (5.47, 34.3) | 13/56 | 23.2 (14.0, 35.9) |
|  |  |  |  |  |  |  |
| *pfdhfr* C59R | 65/80 | 81.3 (71.3, 88.3) | 25/26 | 96.2 (75.9, 99.5) | 40/54 | 74.1 (60.9, 84.0) |
| *pfdhps A437G* | 67/80 | 83.8 (74.1, 90.3) | 22/26 | 84.6 (64.6, 94.3) | 45/54 | 83.3 (71.1, 91.0) |
| *pfdhps* K540E | 2/80 | 2.50 (0.61, 9.62) | 1/26 | 3.85 (0.50, 24.1) | 1/54 | 1.85 (0.25, 12.4) |
| *dhfr + dhps-437* | 57/80 | 71.3 (60.7, 79.9) | 21/26 | 80.8 (60.4, 92.0) | 36/54 | 66.7 (53.6, 77.6) |
| *dhfr + dhps-*437 + *dhps*-540 | 0/80 | 0 | 0/26 | 0 | 0/54 | 0 |
|  |  |  |  |  |  |  |
| **2016** |  |  |  |  |  |  |
| *pfcrt* K76T | 48/100 | 48.0 (38.0, 58.2) | 7/50 | 14.0 (6.70, 27.0) | 41/50 | 82.0 (68.5, 90.5) |
| *pfmdr1* N86Y | 19/100 | 19.0 (12.2, 28.3) | 8/50 | 16.0 (7.53, 30.8) | 11/50 | 22.0 (12.6, 35.6) |
| *pfcrt K76T + pfmdr1* N86Y | 10/100 | 10.0 (5.46, 17.6) | 0/50 | 0 | 10/50 | 20.0 (11.1, 33.4) |
|  |  |  |  |  |  |  |
| *pfdhfr* C59R | 95/97 | 97.9 (92.0, 99.5) | 49/49 | 100 | 46/48 | 95.8 (84.4, 99.0) |
| *pfdhps A437G* | 92/97 | 94.8 (88.1, 97.9) | 48/49 | 98.0 (86.5, 99.7) | 44/48 | 91.7 (79.4, 96.9) |
| *pfdhps* K540E | 3/97 | 3.09 (0.99, 9.25) | 1/49 | 2.04 (0.28, 13.5) | 2/48 | 4.17 (1.01, 15.6) |
| *dhfr + dhps-437* | 90/97 | 92.8 (85.5, 96.5) | 48/49 | 98.0 (86.5, 99.7) | 42/48 | 87.5 (74.5, 94.4) |
| *dhfr + dhps-*437 + *dhps*-540 | 3/97 | 3.09 (0.99, 9.25) | 1/49 | 2.04 (0.28, 13.5) | 2/48 | 4.17 (1.01, 15.6) |

**Table legend**: Prevalence of SP and AQ resistance mutations among study children with *P. falciparum* infection at the end of season surveys in both study centres overall, in Houndé District, Burkina Faso, and in Bougouni District, Mali. Prevalence is calculated as number of resistant mutations / number of children, with mixed mutations at a single locus counted among those resistant. Samples in which both mutant and wild type were detected at two or more codons were excluded.
