# Supplementary material for: Evaluation of seasonal malaria chemoprevention in two areas of intense seasonal malaria transmission: Secondary analysis of a household-randomised, placebo-controlled trial in Houndé District, Burkina Faso and Bougouni District, Mali
Source: PLoS Med. 2020 Aug 21;17(8):e1003214. doi: 10.1371/journal.pmed.1003214 (PMC7442230; doi:10.1371/journal.pmed.1003214)
Supplement: S5 Table — ACPR, adequate clinical and parasitological response; PCR, polymerase chain reaction. (DOCX) [file pmed.1003214.s012.docx]

**S5 Table. Results of 28-day treatment efficacy protocol**

|  |  |  |  | **N** | **% (95% CI)** |
| --- | --- | --- | --- | --- | --- |
| **Burkina Faso** | Total |  |  | 58 |  |
|  |  |  |  |  |  |
|  | ACPR | PCR unadjusted |  | 57 | 98.3 (88.6, 99.8) |
|  |  | PCR adjusted |  | 57 | 98.3 (88.6, 99.8) |
|  |  |  |  |  |  |
|  | Treatment failures | Early treatment failure |  | 1 |  |
|  |  | Late clinical failure |  | 0 |  |
|  |  | Late parasitological failures | Recrudescences | 0 |  |
|  |  |  | Reinfections | 0 |  |
|  |  |  |  |  |  |
| **Mali** | Total |  |  | 153 |  |
|  |  |  |  |  |  |
|  | ACPR | PCR unadjusted |  | 147 | 96.1 (91.5, 98.2) |
|  |  | PCR adjusted |  | 152 | 99.3 (95.4, 99.9) |
|  |  |  |  |  |  |
|  | Treatment failures | Early treatment failure |  | 1 |  |
|  |  | Late clinical failure |  | 0 |  |
|  |  | Late parasitological failure | Recrudescences | 0 |  |
|  |  |  | Reinfections | 5 |  |

**Table legend**: Abbreviations: ACPR, adequate clinical and parasitological response; PCR, polymerase chain reaction.
