# Supplementary material for: Evaluation of seasonal malaria chemoprevention in two areas of intense seasonal malaria transmission: Secondary analysis of a household-randomised, placebo-controlled trial in Houndé District, Burkina Faso and Bougouni District, Mali
Source: PLoS Med. 2020 Aug 21;17(8):e1003214. doi: 10.1371/journal.pmed.1003214 (PMC7442230; doi:10.1371/journal.pmed.1003214)
Supplement: S1 STROBE Checklist — STROBE, Strengthening the Reporting of Observational Studies in Epidemiology. (DOCX) [file pmed.1003214.s013.docx]

STROBE Statement for the following draft manuscript:

**Evaluation of seasonal malaria chemoprevention in two areas of intense seasonal malaria transmission: secondary analysis of a household-randomised, placebo-controlled trial in Houndé District, Burkina Faso and Bougouni District, Mali.**

**Authors**

Matthew Cairns^1^, Issaka Sagara^2^, Issaka Zongo^3^, Irene Kuepfer^4,5^, Ismaila Thera^2^, Frederic Nikiema^3^, Modibo Diarra^2^, Serge R. Yerbanga^3^, Amadou Barry^2^, Amadou Tapily^2^, Samba Coumare^2^, Paul Milligan^1^, Halidou Tinto^3^, Jean Bosco Ouédraogo^3^, Daniel Chandramohan^5^, Brian Greenwood^5^, Abdoulaye Djimde^2^, Alassane Dicko^2^.

**Affiliations**

1. Tropical Epidemiology Group, London School of Hygiene and Tropical Medicine, London, UK

2. Malaria Research and Training Centre, Bamako, Mali

3. Institut de Recherche en Sciences de la Santé, Bobo Dioulasso, Burkina Faso.

4. Swiss Tropical and Public Health Institute, Basel, Switzerland.

5. Faculty of Infectious and Tropical Diseases, London School of Hygiene and Tropical Medicine, London, UK

STROBE Statement—Checklist of items that should be included in reports of ***cohort studies***

|  | Item No | Recommendation | Section / Paragraph Title  (NA if not included) |
| --- | --- | --- | --- |
| **Title and abstract** | 1 | (*a*) Indicate the study’s design with a commonly used term in the title or the abstract | Title |
|  |  | (*b*) Provide in the abstract an informative and balanced summary of what was done and what was found | Abstract (Methods and Findings section) |
| Introduction | | |  |
| Background/rationale | 2 | Explain the scientific background and rationale for the investigation being reported | Introduction (first 3 paragraphs) |
| Objectives | 3 | State specific objectives, including any prespecified hypotheses | Author summary, Methods (Study Procedures) |
| Methods | | |  |
| Study design | 4 | Present key elements of study design early in the paper | Methods (Study Procedures) |
| Setting | 5 | Describe the setting, locations, and relevant dates, including periods of recruitment, exposure, follow-up, and data collection | Methods (Study Procedures), Supplemental Figures S1, S2, S3. |
| Participants | 6 | (*a*) Give the eligibility criteria, and the sources and methods of selection of participants. Describe methods of follow-up | Methods: Study Procedures (first paragraph) |
|  |  | (*b*) For matched studies, give matching criteria and number of exposed and unexposed | NA |
| Variables | 7 | Clearly define all outcomes, exposures, predictors, potential confounders, and effect modifiers. Give diagnostic criteria, if applicable | Outcomes, Exposures: Methods section (3^rd^/4^th^/5^th^/6^th^ paragraphs);  Analytical aspects: Methods section (Analysis paragraphs); Statistical Analysis Plan. |
| Data sources/ measurement | 8* | For each variable of interest, give sources of data and details of methods of assessment (measurement). Describe comparability of assessment methods if there is more than one group | Methods Section (Study Procedures) |
| Bias | 9 | Describe any efforts to address potential sources of bias | Exclusion of AZ group (second paragraph of the Methods section); Restriction of analyses to children with 3 or 4 SMC courses that year (second paragraph of the ‘Analysis’ section of the Methods). |
| Study size | 10 | Explain how the study size was arrived at | Methods, Study Procedures |
| Quantitative variables | 11 | Explain how quantitative variables were handled in the analyses. If applicable, describe which groupings were chosen and why | ‘Analysis’ section of the Methods |
| Statistical methods | 12 | (*a*) Describe all statistical methods, including those used to control for confounding | ‘Analysis’ section of the Methods |
|  |  | (*b*) Describe any methods used to examine subgroups and interactions | The ‘Analysis’ section of the Methods describes how SMC records were used to define time since most recent SMC (for incidence), and receipt of most recent SMC (for prevalence at surveys). |
|  |  | (*c*) Explain how missing data were addressed | ‘Analysis’ section of the Methods. ‘Analysis of Treatment Efficacy of SP plus AQ’ describes how mixed infections were handled in the analysis of resistance markers. |
|  |  | (*d*) If applicable, explain how loss to follow-up was addressed | The first paragraph of the ‘Analysis’ section of the Methods describes how person-time at risk was calculated. |
|  |  | (*e*) Describe any sensitivity analyses | Supplementary figures S5 & S6 (by country), Supplementary Table S3 (Prevalence) and Table S4 (prevalence rather than frequency of resistance), |
| Results | | |  |
| Participants | 13* | (a) Report numbers of individuals at each stage of study—eg numbers potentially eligible, examined for eligibility, confirmed eligible, included in the study, completing follow-up, and analysed | Table 1  Paragraphs 1 and 3 of the Results section. |
|  |  | (b) Give reasons for non-participation at each stage | Table 1 contains number under observation. Reasons for exit are presented in the CONSORT chart for the main paper |
|  |  | (c) Consider use of a flow diagram | Presented in CONSORT chart for main paper, summarised in Table 1 |
| Descriptive data | 14* | (a) Give characteristics of study participants (eg demographic, clinical, social) and information on exposures and potential confounders | First Paragraph of Results Section;  Table 1 |
|  |  | (b) Indicate number of participants with missing data for each variable of interest | Table 1, Table 4, Table 5 |
|  |  | (c) Summarise follow-up time (eg, average and total amount) | Third paragraph of Results Section |
| Outcome data | 15* | Report numbers of outcome events or summary measures over time | Results section , paragraph titled ‘Incidence of malaria hospital admissions or deaths from malaria and uncomplicated clinical malaria’ |
| Main results | 16 | (*a*) Give unadjusted estimates and, if applicable, confounder-adjusted estimates and their precision (eg, 95% confidence interval). Make clear which confounders were adjusted for and why they were included | We present only adjusted incidence and prevalence ratios (presenting both may be confusing in this context, as the effect of adjustment is not the focus of the analysis). |
|  |  | (*b*) Report category boundaries when continuous variables were categorized | Age was categorised by year (Methods section), Time since SMC (Analysis paragraphs of Methods section). |
|  |  | (*c*) If relevant, consider translating estimates of relative risk into absolute risk for a meaningful time period | Absolute rates are presented by week in Figures 2 and 3 |
| Other analyses | 17 | Report other analyses done—eg analyses of subgroups and interactions, and sensitivity analyses | These are presented with the relevant main result (e.g. prevalence ratios for end of season surveys) |
| Discussion | | |  |
| Key results | 18 | Summarise key results with reference to study objectives | Discussion (First paragraph) |
| Limitations | 19 | Discuss limitations of the study, taking into account sources of potential bias or imprecision. Discuss both direction and magnitude of any potential bias | Discussion (Paragraphs 4-7) |
| Interpretation | 20 | Give a cautious overall interpretation of results considering objectives, limitations, multiplicity of analyses, results from similar studies, and other relevant evidence | Discussion (Paragraphs 8 and 9) |
| Generalisability | 21 | Discuss the generalisability (external validity) of the study results | Discussion (Paragraph 9) |
| Other information | | |  |
| Funding | 22 | Give the source of funding and the role of the funders for the present study and, if applicable, for the original study on which the present article is based | Funding Paragraph |
